# Melatonin Engineering M2 Macrophage‐Derived Exosomes Mediate Endoplasmic Reticulum Stress and Immune Reprogramming for Periodontitis Therapy

**DOI:** 10.1002/advs.202302029

**Published:** 2023-07-14

**Authors:** Ya Cui, Shebin Hong, Yunhui Xia, Xiaojing Li, Xiaoya He, Xiangying Hu, Yaxin Li, Xudong Wang, Kaili Lin, Lixia Mao

**Affiliations:** ^1^ Department of Oral and Cranio‐Maxillofacial Surgery Shanghai Ninth People's Hospital, College of Stomatology Shanghai Jiao Tong University School of Medicine National Clinical Research Center for Oral Diseases Shanghai Key Laboratory of Stomatology and Shanghai Research Institute of Stomatology Shanghai 200011 China

**Keywords:** endoplasmic reticulum stress, engineered exosomes, immune reprogramming, macrophage, periodontitis

## Abstract

Periodontitis is a chronic infectious disease caused by bacterial irritation. As an essential component of the host immunity, macrophages are highly plastic and play a crucial role in inflammatory response. An appropriate and timely transition from proinflammatory (M1) to anti‐inflammatory (M2) macrophages is indispensable for treating periodontitis. As M2 macrophage‐derived exosomes (M2‐exos) can actively target inflammatory sites and modulate immune microenvironments, M2‐exos can effectively treat periodontitis. Excessive endoplasmic reticulum stress (ER stress) and unfolded protein response (UPR) are highly destructive pathological characteristics during inflammatory periodontal bone loss. Although melatonin has antioxidant and anti‐inflammatory effects, studies focusing on melatonin ER stress modulation remain limited. This study fabricates engineered M2‐exos loading with melatonin (Mel@M2‐exos) for treating periodontitis. As a result, M2‐exos drive an appropriate and timely macrophage reprogramming from M1 to M2 type, which resolves chronic inflammation and accelerated periodontal healing. Melatonin released from Mel@M2‐exos rescues the osteogenic and cementogenic differentiation capacity in inflammatory human periodontal ligament cells (hPDLCs) by reducing excessive ER stress and UPR. Injectable gelatin methacryloyl (GelMA) hydrogels with sustained‐release Mel@M2‐exos accelerate periodontal bone regeneration in rats with ligation‐induced periodontitis. Taken together, melatonin engineering M2 macrophage‐derived exosomes are promising candidates for inflammatory periodontal tissue regeneration.

## Graphical abstract

Periodontitis is an inflammatory disease caused by bacteria, whose progression is closely associated with immune cells. Melatonin engineering M2 macrophage‐derived exosomes, as a natural nanoplatform, effectively mediate endoplasmic reticulum stress (ER stress) and drive immune reprogramming for treating inflammatory periodontal bone loss. This may yield clinically significant effects, and the design of engineered exosomes could have potential clincial applications.

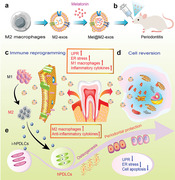

## 1 Introduction

Periodontitis is a chronic infectious disease characterized by the destruction of periodontal tissues, usually causing gum inflammation, peripheral pocket formation, alveolar bone resorption, tooth loosening, and displacement, which is the leading cause of tooth loss in adults.^[^ ^1^ ^]^ In terms of pathogenesis, in addition to pathogenic bacteria,^[^ ^2^ ^]^ the host immune response is also a crucial mediator of periodontal injury.^[^ ^3^ ^]^ Current treatment strategies are mainly based on utilization of periodontal scrapers and antimicrobial drugs to reduce bacterial load, and host modulators can also be used to inhibit the progression of chronic inflammation.^[^ ^4^ ^]^ Nevertheless, alveolar bone loss is a prominent destructive and irreversible feature of periodontal disease, and current treatment options have limited success in preventing ongoing bone loss.^[^ ^5^ ^]^

Macrophages are essential parts of the human innate immune system, which identify, phagocytose, remove bacteria, and foreign bodies.^[^ ^6^ ^]^ Functionally, macrophages are divided into typical (M1), nontypical (M2), and intermediate (M0) types in local tissue microenvironments.^[^ ^7^ ^]^ As the first line of defense against periodontal pathogens, macrophages can kill pathogenic bacteria through their powerful bactericidal phagocytosis.^[^ ^8^ ^]^ However, its excessive activation into M1 type leads to the destruction of periodontal tissues and aggravates the process of periodontitis.^[^ ^9^ ^]^ Indeed, many M1 macrophage‐derived inflammatory factors such as interleukin‐1 (IL‐1) and tumor necrosis factor‐α (TNF‐α) were detected in bone destruction area of periodontitis.^[^ ^10^ ^]^ It is also shown that the amounts of M1‐type macrophages are significantly increased compared with M2‐type macrophages in bone resorption areas of mice with periodontitis, which indicates that there is an imbalance between M1 and M2‐type macrophages in periodontitis, leading to the dominant position of M1‐type macrophages and subsequently alveolar bone resorption.^[^ ^11^ ^]^ Therefore, it is of great significance for chronic inflammation elimination by reversing macrophage polarization and increasing M2‐type macrophages.

Exosomes are naturally occurring nanosized membrane vesicles encased by phospholipid bilayers ranging in size from 30 to 150 nm.^[^ ^12^ ^]^ As a unique class of natural nanomaterials, exosomes can act as mediators of genetic information, carry and deliver biological signals to neighboring and distant cells, regulate the physiological and pathological state of recipient cells, and participate in the progression of various diseases.^[^ ^13^ ^]^ The endogenous and heterogeneous nature of exosomes gives them a broad and unique advantage over synthetic carriers such as liposomes and nanoparticles in the diagnosis and treatment of periodontitis.^[^ ^14^ ^]^ In recent years, several studies have reported the use of macrophage‐derived exosomes as a novel tool for disease monitoring and therapy.^[^ ^15^ ^]^ Macrophages release large amounts of exosomes under both physiological and pathological conditions, encapsulating characteristic contents such as lipids, nucleic acids, and proteins associated with the parent cells, which play a significant role in the transfer of substances and information between cells and enable remotely regulating of the biological functions of target cells.^[^ ^16^ ^]^ Compared to artificial nanocarriers, macrophage‐derived exosomes are safer and easier to modify, which have been extensively investigated for drug delivery and targeted therapy.

Melatonin, a hormone‐like substance produced mainly by the pineal gland, function as effective free radical scavengers, and antioxidants,^[^ ^17^ ^]^ as well as modulate inflammation, and cell apoptosis in different pathophysiological situations.^[^ ^18^ ^]^ It is considered a crucial molecule in cell physiology, which is protective in various cells and tissues.^[^ ^19^ ^]^ For instance, Hu et al. found that melatonin maintained genomic stability, weakened postradiation cortical bone‐derived stem cells (CBSCs) apoptosis and intracellular oxidative stress, and promoted expression of antioxidant‐related enzymes.^[^ ^20^ ^]^ However, high concentration of melatonin accelerates the inflammatory process and induces cell necrosis. Using natural nanoparticles loading with melatonin to achieve controlled drug release may avoid its adverse effects.^[^ ^21^ ^]^ Current studies have demonstrated that melatonin plays a crucial role in the regulation of endoplasmic reticulum stress (ER stress). ER stress refers to the oxidative environment destruction in the lumen of the ER, protein processing, and transport disorders, as well as calcium metabolism disorders, due to lacking of intracellular nutrients, gene mutations, drug stimulation, or infection.^[^ ^22^ ^]^ ER homeostasis is disrupted, protein folding capacity is reduced, and unfolded proteins aggregate, leading to the onset of the unfolded protein response (UPR) .^[^ ^23^ ^]^ Accumulating evidence has illustrated that periodontitis is closely associated with UPR and ER stress. During protein synthesis, the increase of misfolded proteins leads to ER stress, which induces a nonfolded protein response mediated by inositol‐requiring enzyme‐1 (IRE1), protein kinase RNA (PKR)‐like ER kinase (PERK), and activated transcription factor 6 (ATF6) family, when excessive stress will induce cell apoptosis.^[^ ^24^ ^]^ Despite melatonin has been found to alleviate periodontitis, the mechanism involved needs to be clarified. Whether melatonin can suppress excessive ER stress and URP in hPDLCs under the chronic inflammatory microenvironment and rescue periodontal bone loss still needs further investigation.

**Figure 1:**
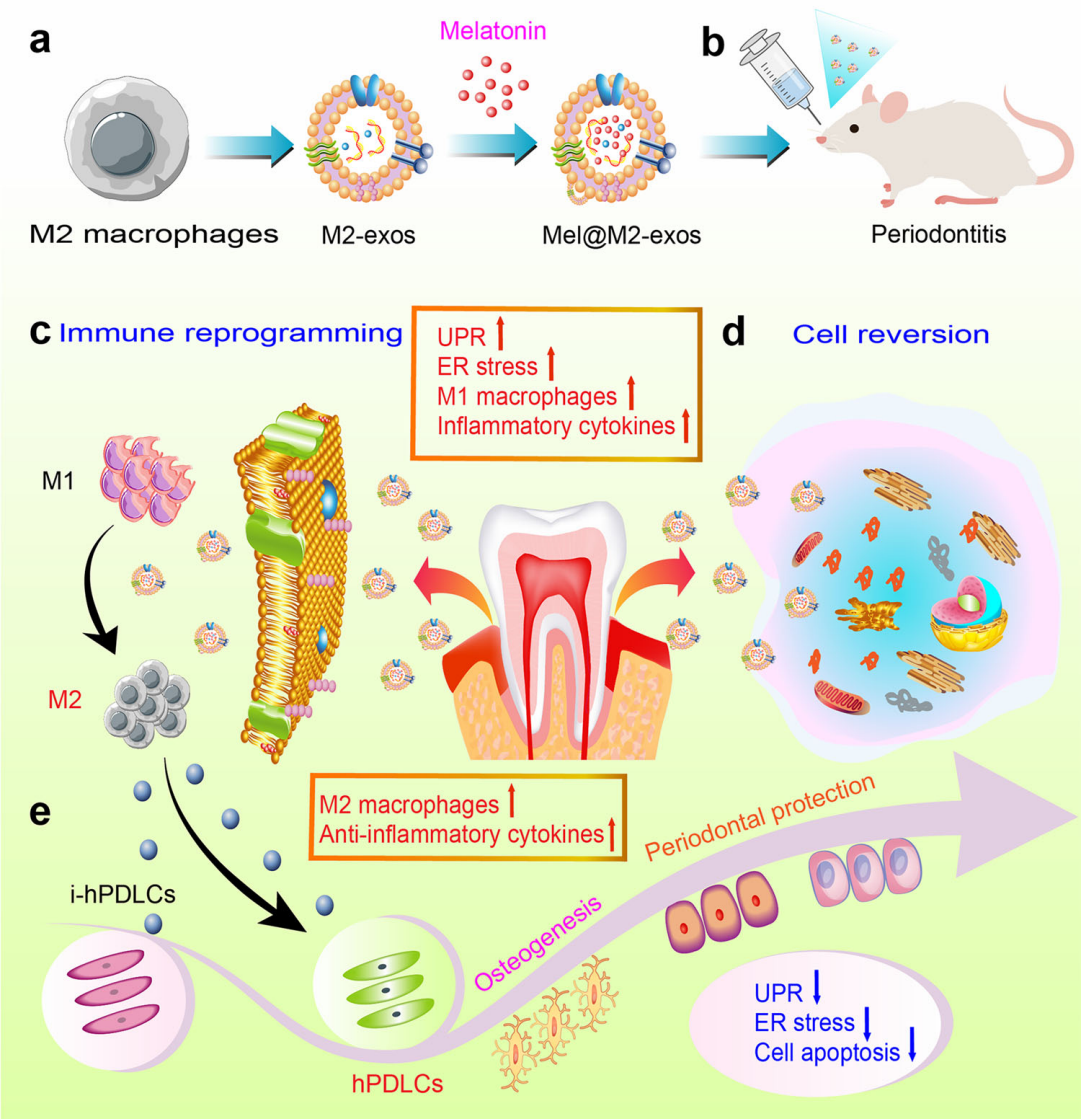
Schematic representation of engineered M2 macrophage‐derived exosomes for treating inflammatory bone loss in periodontitis through mediating ER stress and immune reprogramming. (a) Fabrication of melatonin‐engineered exosomes derived from M2 macrophages. (b) Mel@M2‐exos as a potential treatment for inflammatory bone loss in rats with ligation‐induced periodontitis. (c) Mel@M2‐exos induce immune reprogramming and macrophage repolarization. (d) Mel@M2‐exos mediate endoplasmic reticulum stress and reverse cellular states. (e) Mel@M2‐exos restore the impaired function of hPDLCs under inflammatory conditions.

The main objective of this study was to extract exosomes from M2 macrophages and encapsulate melatonin using intermittent ultrasonic shock, while characterizing their morphology, size, immunophenotype, and other biological features. We further explored whether engineered M2‐exos loading with melatonin can achieve immune reprogramming, induce macrophage polarization in the inflammatory microenvironment, inhibit excessive ER stress, protein misfolding, and cell apoptosis, and rescue the weakened osteogenic, cementogenic, and odontogenic differentiation of hPDLCs under inflammatory states. Meanwhile, injectable GelMA hydrogels with sustained‐release M2‐exos and Mel@M2‐exos were prepared to study the effects of engineered M2 macrophage‐derived exosomes on treating inflammatory periodontal bone loss in ligation‐induced rat model, so as to provide a feasible strategy for the research and development of natural nanomaterials for periodontal bone regeneration (**Figure** **1**).

## 2 Results

### 2.1 The hPDLCs Exhibit Compromised Osteogenic Potential and Excessive ER Stress Under Inflammatory Condition

**Figure 2:**
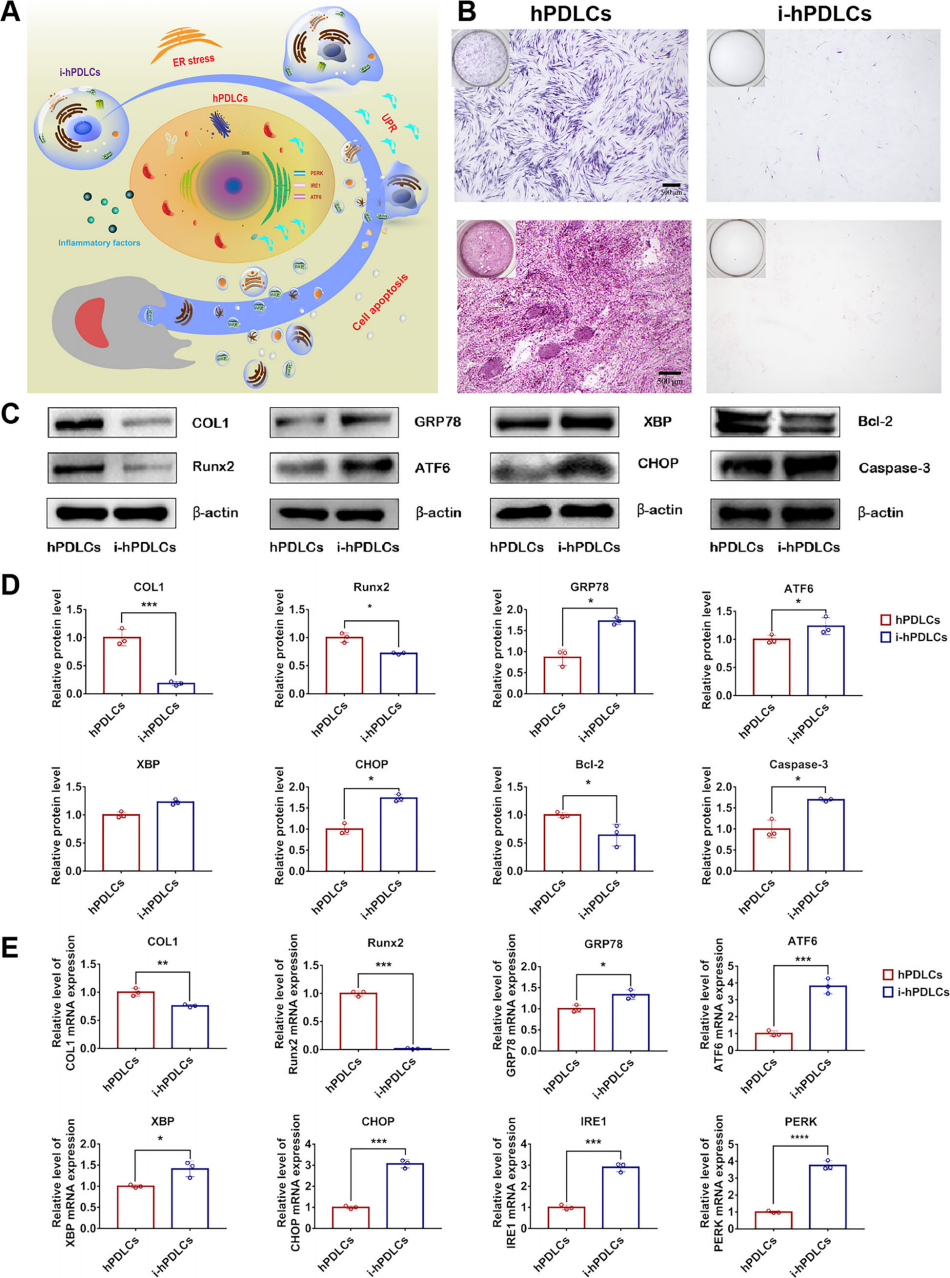
Incubation of human periodontal ligament cells (hPDLCs) under inflammatory conditions leads to compromised osteogenic potential, excessive endoplasmic reticulum stress (ER stress) and cell apoptosis. A) Schematic representation of inflammatory factor inducing ER stress and cell apoptosis in hPDLCs. B) Alkaline phosphatase (ALP) staining, alizarin red staining (ARS), and calcium deposition production in hPDLCs and inflammatory hPDLCs (i‐hPDLCs) (inverted microscope; scale bar: 500 µm). C) Expression of osteogenesis, ER stress, and cell apoptosis‐related proteins (COL1, Runx2, GRP78, ATF6, XBP, CHOP, Bcl‐2, and Caspase‐3) in hPDLCs and i‐hPDLCs (Western blot) and D) quantitative analysis (normalized to β‐actin) in terms of the relative intensity. E) Expression of osteogenesis, and ER stress‐related mRNAs (COL1, Runx2, GRP78, ATF6, XBP, CHOP, PERK, and IRE1) in hPDLCs and i‐hPDLCs (quantitative real‐time polymerase chain reaction (qRT–PCR) assay, normalized to GAPDH). The data are presented as the mean ± SD of *n* = 3. The significance of the data was calculated by the Student's *t*‐test; *p < 0.05, ^**^ *p* < 0.01, and ^***^ *p* < 0.001 indicate significant differences between hPDLCs and i‐hPDLCs.

To investigate the osteogenic differentiation capacity and ER stress levels of periodontal tissues under inflammatory states, the hPDLCs were successfully isolated and purified from human donor teeth and cultured in normal or inflammatory medium with the presence of 20 µg mL^−1^ lipopolysaccharide (LPS). The osteogenic potential of normal and inflammatory hPDLCs (i‐hPDLCs) after a 7‐day prestimulation period were evaluated through alkaline phosphatase (ALP) staining, alizarin red staining (ARS), quantitative real‐time polymerase chain reaction (qRT‐PCR), and Western blot analysis. As a result, hPDLCs tended to produce more ALP (an early osteogenesis‐associated enzyme) and calcium deposition under osteogenic induction conditions (**Figure** **2**B). Further, the expression levels of osteogenic protein and mRNA, including type I collagen (COL1) and runt‐related transcription factor 2 (Runx2), were significantly lower in i‐hPDLCs than in hPDLCs under osteogenic induction conditions (Figure 2C–E). Meanwhile, i‐hPDLCs performed higher ER stress levels under inflammatory states in comparison with hPDLCs. The expression of ER stress related protein, including glucose regulated protein 78 (GRP78), ATF6, X‐box binding protein (XBP), and C/EBP homologous protein (CHOP), as well as mRAN levels of ER stress‐related genes, including GRP78, ATF6, XBP, CHOP, PERK, and IRE1, were significantly higher in i‐hPDLCs than in hPDLCs under inflammatory induction conditions (Figure 2C–E). The expression levels of apoptosis‐related protein Caspase‐3 was upregulated, and B‐cell lymphoma‐2 (Bcl‐2) was downregulated in i‐hPDLCs (Figure 2C,D). On the basis of the foregoing, the inflammatory state induced ER stress and cell apoptosis, and reduced the osteogenic differentiation potential of hPDLCs in periodontal tissue (Figure 2A).

### 2.2 Identification of Engineered M2 Macrophage‐Derived Exosomes with Inflammation Targeting Effects

**Figure 3:**
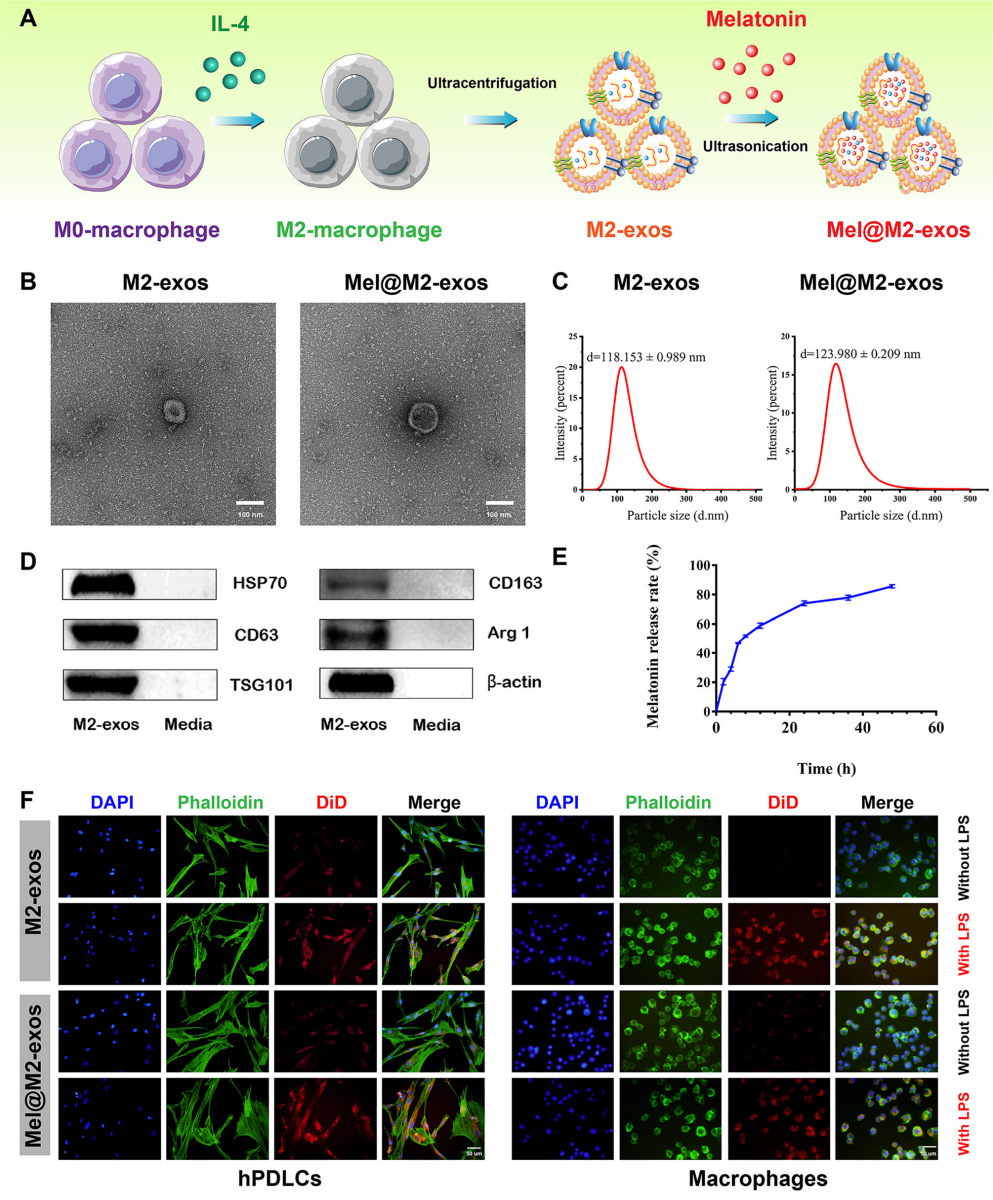
Characterization of M2‐exos and Mel@M2‐exos. A) Schematic representation of the M2‐exos and Mel@M2‐exos synthesis process. B) Transmission electron microscope (TEM) images of M2‐exos and Mel@M2‐exos morphology. Scale bar = 100 nm. C) Representation of particle size analyzed using nanoparticle tracking analysis (NTA). D) Representative Western blots showing exosome markers (CD63, HSP70, and TSG101), M2‐type macrophage markers (CD163, Arg1) and a cytoskeletal protein (β‐actin) of M2‐exos and M2 macrophage conditioned medium. E) The release curves of melatonin from the M2‐exos. F) Uptake of M2‐exos and Mel@M2‐exos by RAW 264.7 cells and human periodontal ligament cells (hPDLCs) with or without lipopolysaccharide (LPS) activation, exosomes were labeled with DiD for red fluorescence.

M2‐exos were isolated from M2 macrophage conditioned medium via ultracentrifugation and identified by transmission electron microscope (TEM), Western blot, and nanoparticle size analysis (NTA). TEM analysis showed that M2‐exos were spherical vesicles with bilayer membrane structure and smooth surface (**Figure** **3**B). NTA showed that the mean diameter of M2‐exos were 118.153 ± 0.989 nm (Figure 3C). Using M2 macrophage supernatant as a control, Western blot analysis showed that M2‐exos expressed CD63, heat shock protein 70 (HSP70), and tumor susceptibility 101 protein (TSG101), which are typically enriched in exosomes. Further Western blot analysis of M2‐exos associated markers arginase 1 (Arg1) and CD163 also showed that these two proteins were enriched in M2‐exos. In addition, the marker β‐actin was also existing in M2‐exos (Figure 3D). The results of the BCA assay showed that approximately 10 µg of M2‐exos (in terms of protein) could be isolated from 10^6^ cells.

Melatonin was successfully loaded into M2‐exos by cyclic ultrasonication with a loading rate of 35.47%. The properties of Mel@M2‐exos were studied by TEM and NTA to investigate the effects of melatonin loading on the physical properties of M2‐exos. TEM analysis showed that drug encapsulation did not affect the morphological structures of M2‐exos, which was a circular nanostructure with similar bilayer membranes and smooth surfaces (Figure 3B). NTA showed that the mean diameters of Mel@M2‐exos were 123.980 ± 0.209 nm (Figure 3C), further indicating that the physical properties of M2‐exos were not affected by the modifications. The release rate of melatonin was about 85.12% at 48 h (Figure 3E). The endocytosis of DiD‐labeled M2‐exos and Mel@M2‐exos by macrophages and hPDLCs (Figure 3F) was observed by confocal laser scanning microscopy (CLSM). The fluorescence signals of M2‐exos and Mel@M2‐exos were detected in both cells, and the combined images show red spots. Interestingly, the fluorescence intensity of M2‐exos and Mel@M2‐exos was significantly higher in LPS‐activated macrophages and hPDLCs than in nonactivated macrophages and hPDLCs. After LPS stimulation, M2‐exos were extensively internalized into macrophages and hPDLCs, indicating a high affinity for target cells in the inflammatory state. These results suggest that M2‐exos have an inherent inflammatory targeting effect, which make them a suitable vehicle for drugs to enter cells under inflammatory states. Taken together, we have successfully prepared M2‐exos and Mel@M2‐exos with potential inflammation and immune‐targeting abilities.

### 2.3 Engineered M2 Macrophage‐Derived Exosomes Accelerate the Osteogenic and Cementogenic Differentiation of hPDLCs Under Inflammatory Conditions

**Figure 4:**
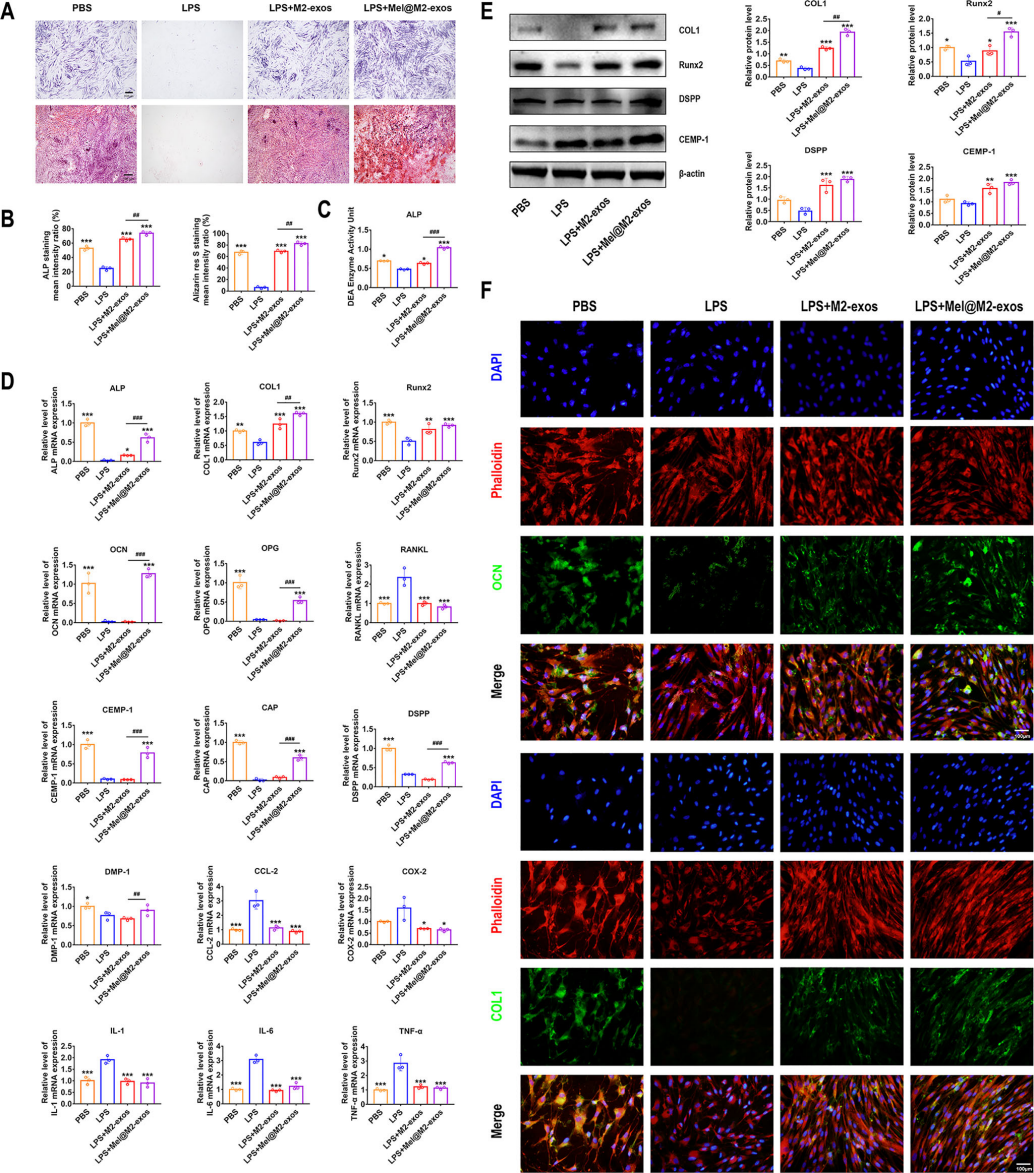
Melatonin engineering M2 macrophage‐derived exosomes enhanced the osteogenic and cementogenic differentiation of human periodontal ligament cells (hPDLCs). A) Alkaline phosphatase (ALP) staining, alizarin red staining (ARS), and calcium deposition production in hPDLCs cultured with phosphate buffer saline (PBS), lipopolysaccharide (LPS), LPS + M2‐exos, LPS + Mel@M2‐exos (inverted microscope; scale bar: 500 µm). B) The quantitative analysis of ALP staining, ARS, and calcium deposition production in hPDLCs cultured with PBS, LPS, LPS + M2‐exos, and LPS + Mel@M2‐exos. C) ALP activity in hPDLCs cultured with PBS, LPS, LPS + M2‐exos, and LPS + Mel@M2‐exos. D) The mRNA levels of osteogenesis, osteoclastogenesis, cementogenesis, odontogenesis, and inflammation related factors (ALP, COL1, Runx2, OCN, OPG, RANKL, CEMP‐1, CAP, DSPP, DMP‐1, CCL‐2, COX‐2, IL‐1, IL‐6, and TNF‐α) in hPDLCs cultured with PBS, LPS, LPS + M2‐exos, and LPS + Mel@M2‐exos (quantitative real‐time polymerase chain reaction (qRT–PCR) assay, normalized to GAPDH). E) Expression of osteogenesis, cementogenesis, and odontogenesis related proteins (COL1, Runx2, DSPP, and CEMP‐1) in hPDLCs cultured with PBS, LPS, LPS + M2‐exos, and LPS + Mel@M2‐exos (Western blot) and quantitative analysis (normalized to β‐actin) in terms of the relative intensity. F) Representative immunofluorescence (IF) images of OCN and COL1 in hPDLCs cultured with PBS, LPS, LPS + M2‐exos, and LPS + Mel@M2‐exos. The data are presented as the mean ± SD of *n* = 3. The significance of the data was calculated by the one‐way analysis of variance (ANOVA); *p < 0.05, ^**^ *p* < 0.01, and ^***^ *p* < 0.001 indicate significant differences with the LPS group; ^#^ *p* < 0.05, ^##^ *p* < 0.01, and ^###^ *p* < 0.001 indicate significant differences between LPS + M2‐exos and LPS + Mel@M2‐exos groups.

Under LPS‐induced inflammatory conditions, we investigated the effects of M2‐exos and Mel@M2‐exos on the osteogenic and cementogenic differentiation of hPDLCs. Under chronic inflammatory microenvironment, the transcript levels of osteogenic factors, including ALP, osteocalcin (OCN), COL1, and Runx2, cementogenic factors, including cementum protein 1 (CEMP‐1), and cementum attachment protein (CAP), as well as odontogenic factors, including dentin phosphophoryn (DSPP) and dentin matrix protein 1 (DMP‐1) were significantly downregulated in hPDLCs (**Figure** **4**D). Similarly, in the ALP and ARS staining results, ALP activity was reduced in hPDLCs under inflammatory conditions. LPS inhibited the formation of mineralized nodules in hPDLCs (Figure 4A,B). Meanwhile, the application of M2‐exos to hPDLCs under inflammatory conditions accelerated the osteogenic process. In M2‐exos stimulated inflammatory hPDLCs group (LPS + M2‐exos), the mRNA levels of ALP, COL1, and Runx2 (Figure 4D), ALP activity (Figure 4C), as well as COL1 and Runx2 protein levels (Figure 4E) were elevated. However, the mRNA level of OCN did not change significantly. In addition, ALP and ARS staining (Figure 4A,B) also showed higher levels of ALP and calcium deposition production when M2‐exos were used in inflammatory hPDLCs. For the cementogenic and odontogenic factors, there were no significant changes in the mRNA levels of CEMP‐1, CAP, DSPP, and DMP‐1 in the LPS + M2‐exos group compared to the LPS group (Figure 4D).

Furthermore, Mel@M2‐exos further enhanced the osteogenic differentiation of hPDLCs under the inflammatory states compared to M2‐exos. In Mel@M2‐exos stimulated inflammatory hPDLCs group (LPS + Mel@M2‐exos), the mRNA levels of ALP, OCN, COL1 (Figure 4D), ALP activity (Figure 4C), as well as COL1 and Runx2 protein levels (Figure 4E) were significantly higher than in the LPS + M2‐exos group. However, the mRNA level of Runx2 did not change significantly. In addition, ALP and ARS staining (Figure 4A,B) also showed a proximal increasing in the levels of ALP and calcium deposition production when Mel@M2‐exos was used in inflammatory hPDLCs compared to M2‐exos. The immunofluorescence (IF) images also showed an increase of OCN and COL1‐labeled hPDLCs after cultured with M2‐exos and Mel@M2‐exos, with the most pronounced improvement in the Mel@M2‐exos group (Figure 4F). For the specific cementogenic and odontogenic factors, the mRNA levels of CEMP‐1, CAP, DSPP and DMP‐1 were increased but not restored to normal with the addition of Mel@M2‐exos into inflammatory hPDLCs (Figure 4D). Meanwhile, M2‐exos and Mel@M2‐exos also inhibited the mRNA levels of inflammation related genes, including C‐C motif chemokine 2 (CCL‐2), cyclooxygenase 2 (COX‐2), interleukin‐6 (IL‐6), IL‐1, and TNF‐α (Figure 4D).

In addition, the expression of receptor activator nuclear factor kappa B ligand (RANKL) and osteoprotegerin (OPG), factors associated with osteoclastic activity, was detected in hPDLCs. We found that the mRNA level of OPG was downregulated in the inflammatory microenvironment (the LPS group). M2‐exos did not increase the production of OPG, whereas Mel@M2‐exos upregulated the mRNA level of OPG (Figure 4D). Meanwhile, the application of M2‐exos and Mel@M2‐exos induced a decrease of RANKL in inflammatory hPDLCs (Figure 4D). In contrast to the LPS group, although M2‐exos inhibited the RANKL/OPG ratio of hPDLCs under inflammatory microenvironment, the inhibitory effect of Mel@M2‐exos on the RANKL/OPG ratio was more pronounced with the combined effects of melatonin and M2‐exos (Figure 4D). On the basis of above data, we found that Mel@M2‐exos could effectively improve osteogenic, cementogenic, and odontogenic activity, as well as reduce osteoclastic activity, indicating that Mel@M2‐exos have great value and potential for periodontal regeneration in vitro.

### 2.4 Engineered M2 Macrophage‐Derived Exosomes Restore the Impaired Function of hPDLCs Under Inflammatory Conditions Via Reducing Excessive ER Stress

**Figure 5:**
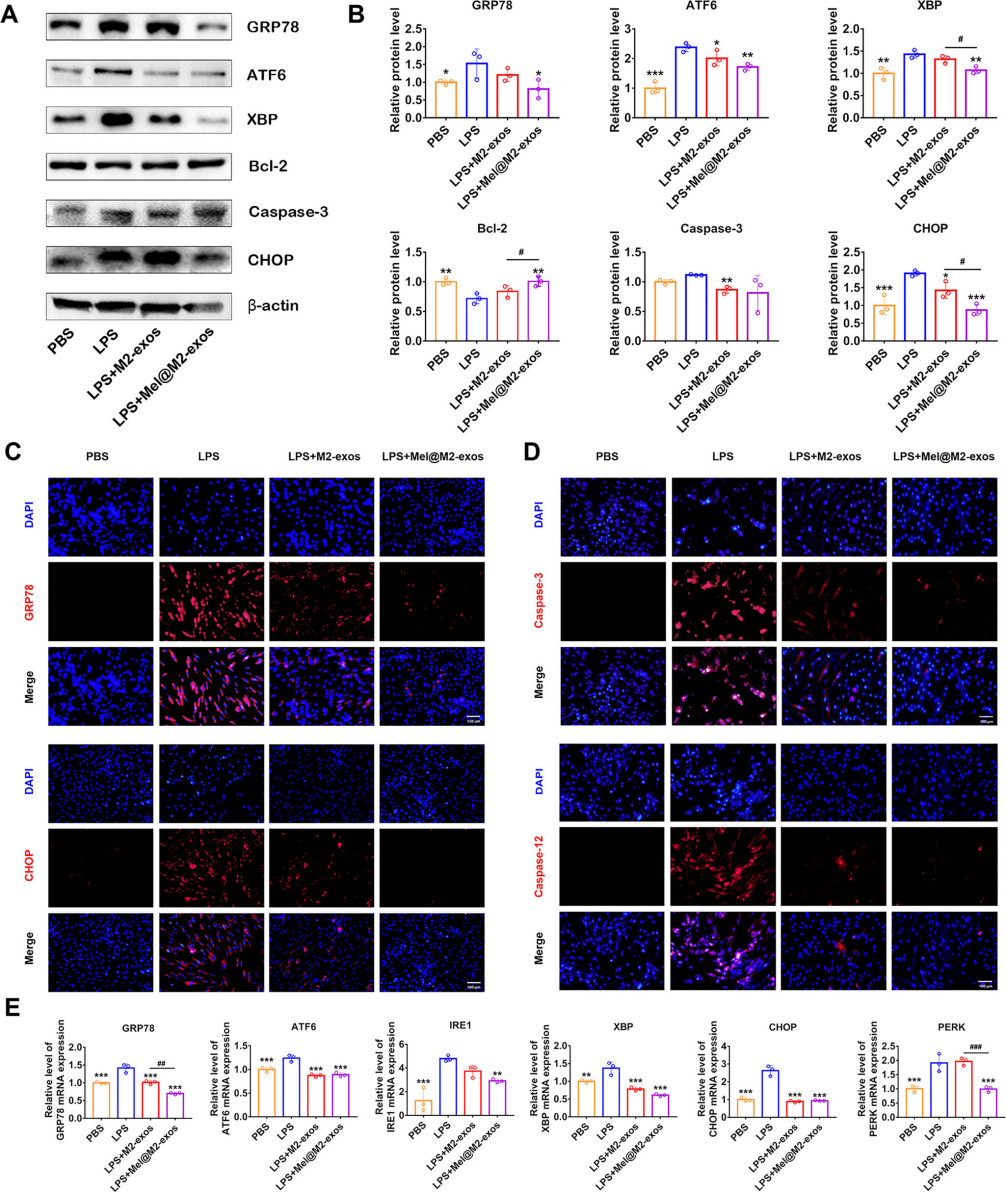
Melatonin engineering M2 macrophage‐derived exosomes restore the impaired function of human periodontal ligament cells (hPDLCs) under inflammatory conditions by reducing excessive endoplasmic reticulum stress (ER stress) and apoptosis. A) Expression of ER stress and apoptosis protein (GRP78, ATF6, XBP, Bcl‐2, Caspase‐3, and CHOP) in hPDLCs cultured with phosphate buffer saline (PBS), lipopolysaccharide (LPS), LPS + M2‐exos, LPS + Mel@M2‐exos (Western blot), and B) quantitative analysis (normalized to β‐actin) in terms of the relative intensity. C) Representative immunofluorescence (IF) images of GRP78 and CHOP in hPDLCs cultured with PBS, LPS, LPS + M2‐exos, and LPS + Mel@M2‐exos. Nuclei were stained with DAPI. Scale bar = 100 µm. D) Representative IF images of Caspase‐3 and Caspase‐12 in hPDLCs cultured with PBS, LPS, LPS + M2‐exos, and LPS + Mel@M2‐exos. Nuclei were stained with DAPI. Scale bar = 100 µm. E) Expression of ER stress‐related mRNAs (GRP78, ATF6, XBP, CHOP, PERK, and IRE1) in hPDLCs cultured with PBS, LPS, LPS + M2‐exos, and LPS + Mel@M2‐exos (quantitative real‐time polymerase chain reaction (qRT–PCR) assay, normalized to GAPDH). The data are presented as the mean ± SD of *n* = 3. The significance of the data was calculated by the one‐way analysis of variance (ANOVA); *p < 0.05, ^**^ *p* < 0.01, and ^***^ *p* < 0.001 indicate significant differences with the LPS group; ^#^ *p* < 0.05, ^##^ *p* < 0.01, and ^###^ *p* < 0.001 indicate significant differences between LPS + M2‐exos and LPS + Mel@M2‐exos groups.

Indeed, inflammation is thought to have deleterious effects on cells by inducing severe ER stress and UPR. To assess the effects of engineered M2 macrophage‐derived exosomes on hPDLCs, we treated hPDLCs with M2‐exos and Mel@M2‐exos, respectively, under LPS stimulation. In the inflammatory microenvironment, the transcript levels of GRP78, a marker of ER stress, were significantly upregulated in hPDLCs (**Figure** **5**E). Meanwhile, the application of M2‐exos to hPDLCs under inflammatory condition suppressed the ER stress. In LPS + M2‐exos group, the mRNA level of GRP78 (Figure 5E) was reduced, and Mel@M2‐exos further reduced the level of ER stress in hPDLCs under inflammatory state compared to M2‐exos. In LPS + Mel@M2‐exos group, the mRNA level of GRP78 (Figure 5E) was significantly lower than that in the LPS + M2‐exos group. The expression levels of GRP78 were then analyzed via Western blot, and quantitative protein levels were calculated (Figure 5A,B). IF analysis also showed an increase of GRP78‐labeled and CHOP‐labeled hPDLCs in the LPS group and a significant decrease of GRP78‐labeled and CHOP‐labeled cells when combined with M2‐exos and Mel@M2‐exos, with the most pronounced reduction in the Mel@M2‐exos group (Figure 5C). These experimental results suggest that melatonin, combined with M2‐exos can reduce LPS‐induced ER stress in hPDLCs. In particular, one of the responses available to cells during ER stress is apoptosis, which is usually the preferred reaction of cells when other control mechanisms are overwhelmed.^[^ ^25^ ^]^ As severe ER stress leads to cell apoptosis,^[^ ^26^ ^]^ we next compared the activation levels of Caspase‐3 and Caspase‐12 among different groups. The Caspase‐3‐labeled or Caspase‐12‐labeled cells were increased in the LPS treatment group and decreased in combination with M2‐exos and Mel@M2‐exos (Figure 5D). Taken together, our study found that LPS increased ER stress in hPDLCs, and that Mel@M2‐exos therapy may exert a protective effect by reducing ER stress‐induced apoptosis.

Previous studies have shown that severe or prolonged ER stress can induce excessive activation of the UPR, leading to excessive protein degradation and ultimately cell death. To further investigate the role of Mel@M2‐exos in LPS‐induced ER stress, we determined whether Mel@M2‐exos treatment could modulate UPR activation, and cell apoptosis. RT‐qPCR examined three transmembrane proteins in UPR (Figure 5E). As expected, LPS induced the increased expression of PERK, ATF6, IRE1, and XBP, indicating activation of the ER stress and UPR in hPDLCs. In addition, CHOP, a mediator of ER stress‐related apoptosis, was activated at the transcriptional and protein levels under LPS induction (Figure 5A,E). Interestingly, the expression of cell apoptosis related protein Caspase‐3 was significantly reduced in the LPS + Mel@M2‐exos group compared to the LPS group, whereas Bcl‐2 was upregulated (Figure 5A,B). In conclusion, these data demonstrate that Mel@M2‐exos regulate UPR activation in response to LPS‐induced ER stress in hPDLCs.

### 2.5 Engineered M2 Macrophage‐Derived Exosomes Mediate Immune Reprogramming and Macrophage Polarization from a Proinflammatory Phenotype to an Anti‐Inflammatory Phenotype

**Figure 6:**
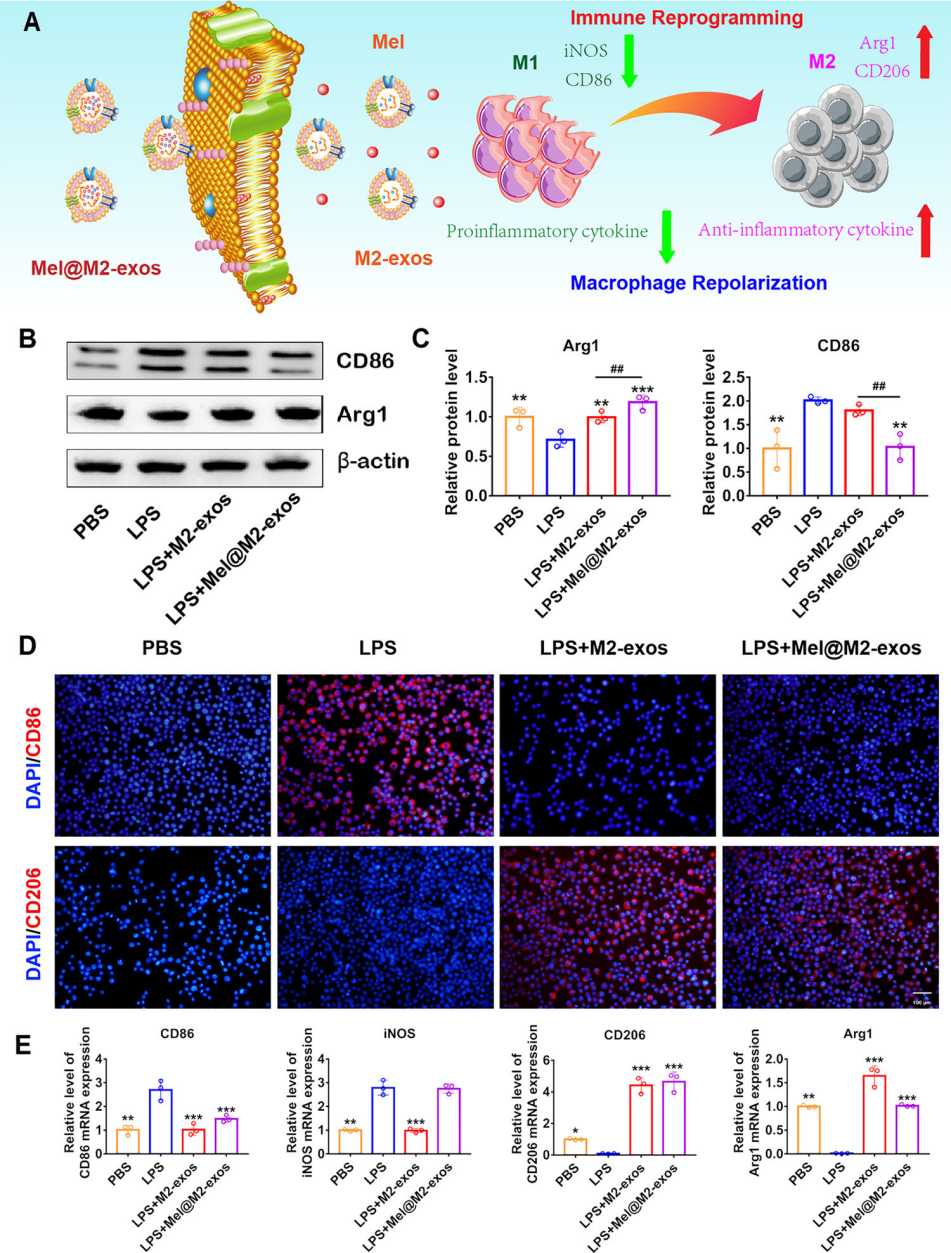
Melatonin engineering M2 macrophage‐derived exosomes mediate immune reprogramming and macrophage polarization from a proinflammatory phenotype to an anti‐inflammatory phenotype. A) Schematic representation of engineered M2 macrophage‐derived exosomes reprogramming inflammation‐associated macrophages into M2‐like macrophages. B) Expression of macrophage polarization‐related protein (CD86 and Arg1) in RAW 264.7 cells cultured with phosphate buffer saline (PBS), lipopolysaccharide (LPS), LPS + M2‐exos, LPS + Mel@M2‐exos (Western blot), and C) quantitative analysis (normalized to β‐actin) in terms of the relative intensity. D) Representative immunofluorescence (IF) images of CD86^+^ and CD206^+^ cells in RAW 264.7 cells cultured with PBS, LPS, LPS + M2‐exos, and LPS + Mel@M2‐exos. Nuclei were stained with DAPI. Scale bar = 100 µm. E) The mRNA levels of macrophage polarization related factors (CD86, iNOS, CD206, and Arg1) in RAW 264.7 cells cultured with PBS, LPS, LPS + M2‐exos, and LPS + Mel@M2‐exos (quantitative real‐time polymerase chain reaction (qRT–PCR), normalized to GAPDH). The data are presented as the mean ± SD of *n* = 3. The significance of the data was calculated by the one‐way analysis of variance (ANOVA); *p < 0.05, ^**^ *p* < 0.01, and ^***^ *p* < 0.001 indicate significant differences with the LPS group; ^#^ *p* < 0.05, ^##^p < 0.01, and ^###^ *p* < 0.001 indicate significant differences between LPS + M2‐exos and LPS + Mel@M2‐exos groups.

Macrophages play an important role in periodontal tissue repair through participating in the phagocytosis of tissue debris and reducing inflammatory response. Their conversion from a proinflammatory phenotype to an anti‐inflammatory phenotype is significant for the resolution of inflammation. To determine whether M2‐exos and Mel@M2‐exos therapy polarizes macrophages from the M1 to the M2 states, we quantified the expression of M1‐specific markers, including CD86 and inducible nitric oxide synthase (iNOS), as well as M2‐specific markers, including CD206 and Arg1, in the presence of phosphate buffer saline (PBS), LPS, M2‐exos and Mel@M2‐exos. IF and Western blotting analysis showed that LPS (50 ng mL^−1^) increased the protein expression of CD86 and Arg1, as well as decreased the protein expression of CD206 in macrophages (**Figure** **6**B–D). Meanwhile, RT‐qPCR results showed that LPS upregulated the mRNA levels of CD86 and iNOS, while downregulated the mRNA levels of CD206 and Arg1 (Figure 6E), which indicated that M1‐type macrophages may predominate in the inflammatory microenvironments. It is worth noting that M2‐exos and Mel@M2‐exos decreased the protein expression of CD86 and increased the protein expression of CD206 (Figure 6B–D). Further, M2‐exos significantly downregulated the mRNA levels of CD86 and iNOS and upregulated the mRNA levels of CD206 and Arg1 (Figure 6E). Mel@M2‐exos also downregulated the mRNA level of CD86 and upregulated the mRNA level of CD206, but had no significant effects on the mRNA levels of iNOS and Arg1 (Figure 6E). Taken together, these results suggest that M2‐exos promote the conversion of macrophages from a proinflammatory phenotype to an anti‐inflammatory phenotype.

### 2.6 Injectable GelMA Hydrogels Incorporated with Melatonin Engineering M2 Macrophage‐Derived Exosomes Reverse Alveolar Bone Loss in a Rat Model of Periodontitis Induced by Ligation

**Figure 7:**
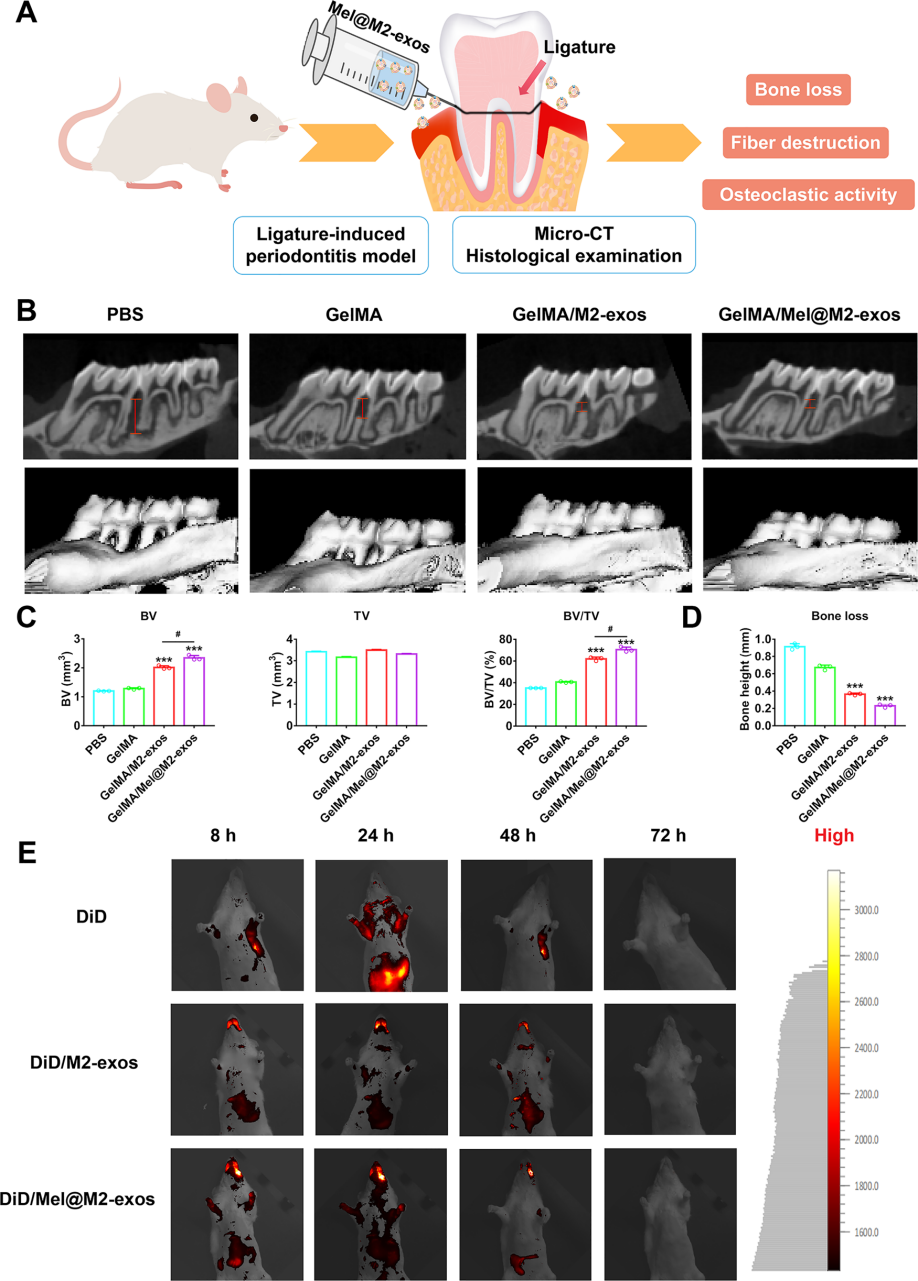
Mel@M2‐exos inhibited bone loss in rat ligature‐induced periodontitis. A) Schematic diagram of the rat periodontitis experiment. B) 3D reconstructed digitized images, and mesiodistal section images of the maxillary first and second molars analyzed by micro‐CT. C) Bone‐related parameters (BV, TV, and BV/TV) and D) bone height between maxillary first and second molars in different groups analyzed by micro‐CT. E) Imaging of SD rat with ligature‐induced periodontitis at different time points after intravenous injection with free DiD, DiD/M2‐exos, and DiD/Mel@M2‐exos. The data are presented as the mean ± SD of *n* = 3. The significance of the data was calculated by the one‐way analysis of variance (ANOVA); *p < 0.05, ^**^ *p* < 0.01, and ^***^ *p* < 0.001 indicate significant differences with the phosphate buffer saline (PBS) group.

Given the positive effects of M2‐exos and Mel@M2‐exos on inflammatory regulation and the rescue of hPDLCs, we conducted in vivo studies to further confirm the interventional effects of M2‐exos and Mel@M2‐exos on inflammatory periodontal bone loss. As shown schematically (**Figure** **7**A), we successfully prepared GelMA hydrogels loading with M2‐exos and Mel@M2‐exos, respectively, and injected them into the gingival sulcus between maxillary first and second molars in rats with ligature‐induced periodontitis. Based on micro‐CT analysis, 3D reconstructed images, and cross‐sectional images are shown in Figure 7B, while BV, TV, BV/TV, and bone height were measured (Figure 7C,D). We observed that ligation successfully induced periodontitis in the rats and resulted in bone loss, and reduced bone height around the teeth (the PBS group). Injections of PBS and GelMA into the gingival sulcus between maxillary first and second molars failed to alleviate periodontal bone loss. However, injections of M2‐exos and Mel@M2‐exos significantly alleviated periodontal bone loss, with the GelMA + Mel@M2‐exos group being the most effective. To further validate the targeting of M2‐exos and Mel@M2‐exos on inflammatory lesions and macrophages, we constructed a ligation‐induced rat periodontitis model. DiD‐labeled M2‐exos and Mel@M2‐exos systems were injected into the model rats for in vivo imaging assays. The results showed that M2‐exos and Mel@M2‐exos were enriched in the vicinity of the rat periodontal bone, demonstrating the in vivo inflammatory defect targeting of M2‐exos and Mel@M2‐exos. Further results showed that the local enrichment of M2‐exos and Mel@M2‐exos in inflamed periodontal bone started at 8 h after injection, peaked at 24 h and then gradually declined until it disappeared after 72 h. This demonstrated the excellent in vivo targeting of M2‐exos and Mel@M2‐exos, increasing their retention time at the target site, effectively increasing their efficacy and reducing off‐target effects and adverse effects.

**Figure 8:**
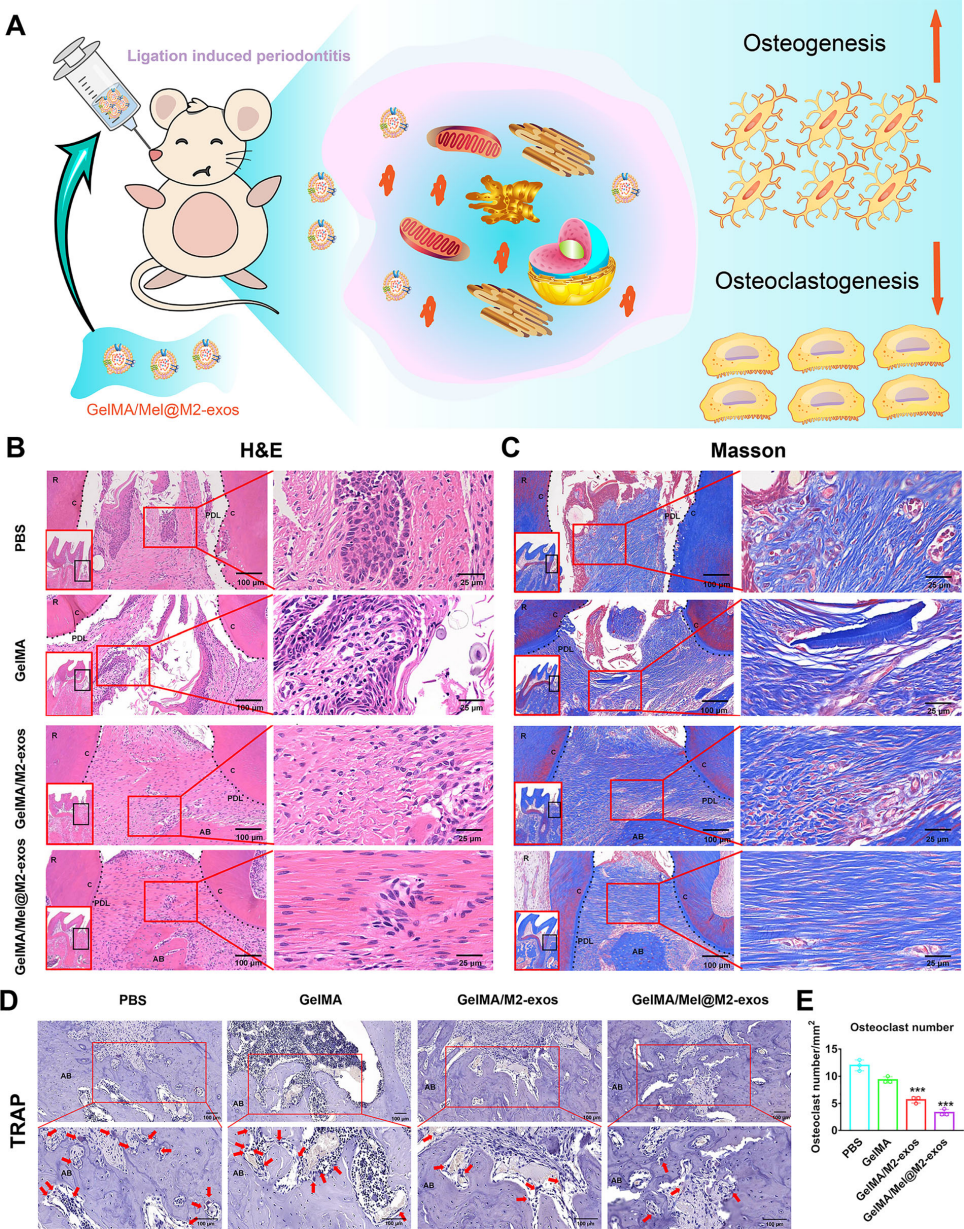
Mel@M2‐exos reduced the periodontal destruction in rat ligature‐induced periodontitis. A) Schematic diagram of engineered M2 macrophage‐derived exosomes promoting osteogenesis and inhibiting osteoclastogenesis. B) Hematoxylin‐eosin (H&E) staining, C) Masson staining, and D) tartaric acid phosphatase (TRAP) staining images (arrows indicate osteoclasts that are dyed red), and E) the corresponding quantitative analysis of the osteoclast number in periodontal tissues. The data are presented as the mean ± SD of *n* = 3. The significance of the data was calculated by the one‐way analysis of variance (ANOVA); *p < 0.05, ^**^ *p* < 0.01, and ^***^ *p* < 0.001 indicate significant differences with the phosphate buffer saline (PBS) group. AB = alveolar bone; C = cementum; PDL = periodontal ligament.

Hematoxylin‐eosin (H&E) and Masson staining (**Figure** **8**B,C) showed that in the GelMA/M2‐exos and GelMA/Mel@M2‐exos treated groups, the periodontal tissue had a thicker epithelial cell layer, a lower number of infiltrating inflammatory cells, denser and more organized elastic and collagen fibers, and more alveolar bone. In contrast, in the PBS and GelMA groups, inflammation led to fibrous degeneration and degradation, subsequently resulting in a disorganized and sparse arrangement of fibers in the periodontal tissue and a reduction in the amount of alveolar bone. Meanwhile, the cementum and even dentin in PBS and GelMA groups, marked by black dashed line, had been resorbed due to inflammation. Nevertheless, the utilization of Mel@M2‐exos and M2‐exos protected the cementum from inflammatory invasion, and left Sharpey fibres and cementum almost intact (Figure 8B,C; Figure S5, Supporting Information). Furthermore, anti‐tartaric acid phosphatase (TRAP) staining and quantitative analysis (Figure 8D,E; Figure S4, Supporting Information) showed that GelMA/M2‐exos and GelMA/Mel@M2‐exos reduced the number of osteoclasts when ligation was present, with GelMA/Mel@M2‐exos being more effective.

**Figure 9:**
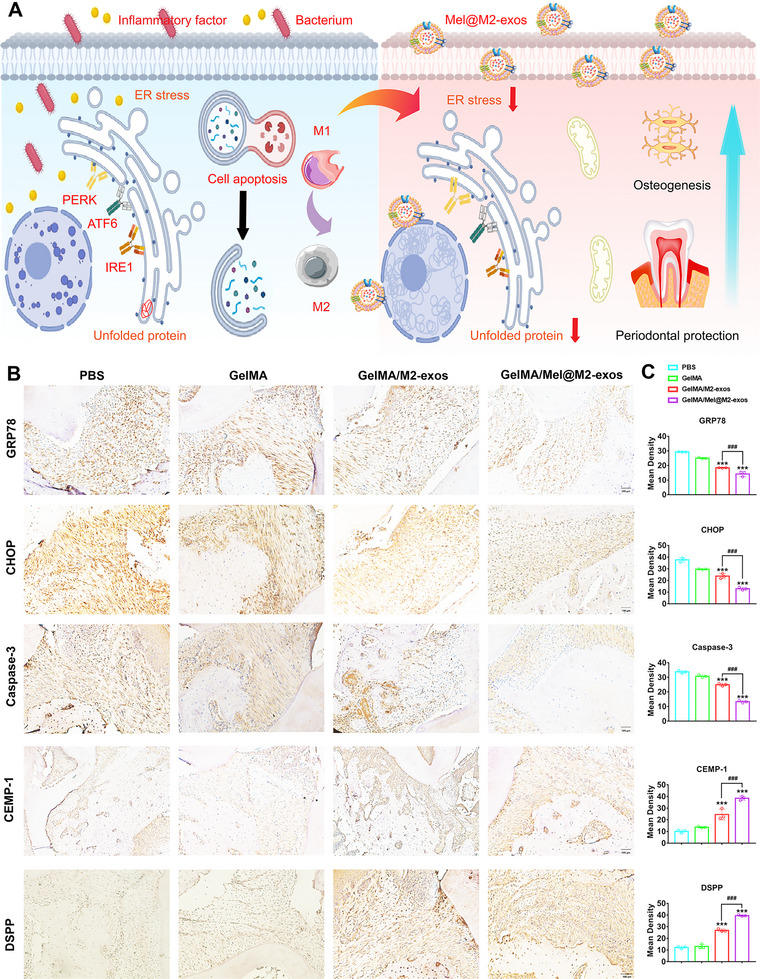
Mel@M2‐exos inhibited the expression of endoplasmic reticulum stress (ER stress) and cell apoptosis related proteins, increased the expression of cementogenic and odontogenic proteins. A) Schematic diagram of engineered M2 macrophage‐derived exosomes inhibiting ER stress and cell apoptosis for periodontitis treatment. B) Expression of ER stress and apoptosis‐related proteins (GRP78, CHOP, and Caspase‐3), as well as cementogenic and odontogenic proteins (CEMP‐1 and DSPP) in phosphate buffer saline (PBS), GelMA, GelMA + M2‐exos, and GelMA + Mel@M2‐exos groups and C) quantitative analysis. The data are presented as the mean ± SD of *n* = 3. The significance of the data was calculated by the one‐way analysis of variance (ANOVA); *p < 0.05, ^**^ *p* < 0.01, and ^***^ *p* < 0.001 indicate significant differences with the PBS group. AB = alveolar bone; PDL = periodontal ligament.

The immunohistochemistry (**Figure** **9**B) and quantitative analysis (Figure 9C) showed that PBS and GelMA groups expressed more ER stress and cell apoptosis protein, including GRP78, CHOP, and Caspase‐3. The expression of GRP78, CHOP, and Caspase‐3 in periodontal tissues was significantly decreased after GelMA/M2‐exos and GelMA/Mel@M2‐exos therapy. Furthermore, the expression of cementogenic and odontogenic proteins, including CEMP‐1 and DSPP, was reduced with injection of PBS and GelMA. Interestingly, GelMA/M2‐exos and GelMA/Mel@M2‐exos therapy improved the expression of CEMP‐1 and DSPP, with the best effect in the GelMA/Mel@M2‐exos group. These results indicate that the infusion of GelMA/Mel@M2‐exos effectively alleviates experimental periodontitis in rats and rescues alveolar bone loss.

## 3 Discussion

The imbalance of macrophage polarization and high levels of ER stress are key factors in the persistent progression of chronic periodontal inflammation, unmatched osteogenic/osteoclastic homeostasis, and subsequently bone defects.^[^ ^27^ ^]^ The expression of proinflammatory factors TNF‐α and IL‐1 were found to be increased in the periodontal pockets of mice with periodontitis.^[^ ^28^ ^]^ Further, M1‐type macrophages stimulated the activation of T cells and polymorphonuclear neutrophils, leading to alveolar bone loss.^[^ ^29^ ^]^ Indeed, the imbalance in the M1/M2 macrophage ratio is an important cause of dysregulation in the host immune inflammatory response and periodontal tissue destruction.^[^ ^30^ ^]^ Our study found that the mRNA and protein levels of ER stress‐related factors were increased in hPDLCs after LPS induction. The protein levels of cell apoptosis‐factors Caspase‐3 were increased, whereas the protein levels of Bcl‐2 were decreased in i‐hPDLCs. Meanwhile, the mRNA and protein levels of osteogenesis‐related factors were decreased, and ALP activity and calcium nodule formation were reduced in i‐hPDLCs. To some extent, the inflammatory microenvironment caused by macrophage polarization imbalance may induce excessive ER stress, UPR, and cell apoptosis in hPDLCs. In this article, we have prepared exosomes derived from M2 macrophage that effectively encapsulate melatonin. M2‐exos specifically targeted inflammatory regions and mediated immune reprogramming to polarize macrophages from M1 to M2 type. At the same time, the sequential release of melatonin, in concert with M2‐exos, exerted osteoprotective effects, regulated high levels of intracellular ER stress and UPR, inhibited cell apoptosis, restored the potential of osteogenic and cementogenic differentiation in hPDLCs, and ultimately accelerated periodontal regeneration.

Immune remodeling is one of the most promising options for treating periodontal inflammation and promoting tissue repair.^[^ ^31^ ^]^ The excessive activation of macrophages is one of the earliest inflammatory responses following periodontal injury. Many proinflammatory factors are released into the damaged microenvironment and mononuclear macrophages are recruited to the site of chronic inflammation, creating an uncontrollable inflammatory response.^[^ ^32^ ^]^ Macrophages that accumulate at bone defect sites in the early stages of inflammation are predominantly of the M1 type, indicating a severe imbalance of macrophage polarization in response to proinflammatory factors.^[^ ^33^ ^]^ Therefore, it is a potential therapeutic strategy for uncontrollable inflammatory responses by regulating the polarization of macrophages from M1 into M2 macrophages in periodontitis. We have found that M2‐exos can actively target inflammatory sites and exert the effects of inflammatory inhibition. There was strong molecular recognition among M2‐exos, activated RAW 264.7 cells and hPDLCs, which resulted in greater drug uptake by LPS‐activated macrophages and hPDLCs than nonactivated macrophages and hPDLCs, ultimately leading to selective drug accumulation in the inflamed periodontal pockets. Meanwhile, the uptake of M2‐exos by LPS‐activated macrophages resulted in a decrease in the initially highly expressed M1‐specific markers (iNOS, CD86) and an increase in the lowly expressed M2‐specific markers (CD206, Arg1), suggesting that M2‐exos can polarize M1‐type into M2‐type macrophages after taken up by activated macrophages. Li et al. also found that the anti‐inflammatory effects of M2‐exos were attributed to specific interactions between lymphocyte function‐associated antigen 1 (LFA‐1) and very late activation 4 (VLA‐4) on their surfaces and intercellular adhesion molecule 1 (ICAM‐1) and receptor in LPS‐activated macrophages, allowing them to target inflammatory joints.^[^ ^34^ ^]^ In summary, M2‐exos can be used as a simple and effective drug carrier with inflammatory targeting and anti‐inflammatory properties for treating periodontitis.

The ER stress pathway, in particular, is closely associated with cell apoptosis in inflammatory hPDLCs.^[^ ^35^ ^]^ The ER is one of the critical organelles and plays a significant role in protein synthesis, folding, and maturation. Especially, the ER stress results from disturbances in Ca^2+^ homeostasis and the accumulation of unfolded or misfolded proteins in the lumen of the ER.^[^ ^36^ ^]^ Under normal conditions, ER stress restores the homeostasis of ER through increasing the capacity of protein folding and activating UPR. Furthermore, PERK, ATF6, and IRE1 are three ER‐localized transmembrane proteins that act as sensors for misfolded proteins in the lumen of the ER and are covered by GRP78. However, in the presence of misfolded proteins, these chaperones are separated and the corresponding UPR signaling pathway is activated, thereby promoting cell apoptosis via the ER stress‐specific proapoptotic factor, CHOP.^[^ ^37^ ^]^ In this study, the expression of ER stress‐related proteins (GRP78, ATF6, PERK, IRE1, XBP, and CHOP) was increased in the LPS group, suggesting that high concentrations of LPS can activate the ER stress signaling pathway and cause an increase in the expression of related proteins. Indeed, the caspase family plays a crucial role in apoptosis and works with several factors to regulate cell apoptosis.^[^ ^38^ ^]^ Activated PERK, IRE1, and ATF6 enhance the transcriptional activity of Caspase‐3 by upregulating the downstream molecule CHOP. High expression of Caspase‐3 mediates the apoptotic cascade response and promotes cell apoptosis.^[^ ^39^ ^]^ Furthermore, the disruption of Ca^2+^ homeostasis in the ER has been found to trigger ER stress, leading to the release of apoptotic factors such as cytochrome c (Cyt c) from the mitochondrial membrane, thereby activating the caspase apoptotic signaling pathway.^[^ ^40^ ^]^ The results of this study suggest that high concentrations of LPS can induce cell apoptosis in hPDLCs.

Melatonin is a hormone‐like substance produced mainly by the pineal gland and has antioxidant, anti‐inflammatory, and wound healing effects.^[^ ^41^ ^]^ It has been found that melatonin alleviated periodontitis and exerted osteoprotective effects. Since high concentrations of melatonin accelerate the inflammatory process and induce cell necrosis, using natural nanoparticles loading with melatonin allows for its controlled release and prevents adverse reactions.^[^ ^42^ ^]^ We found that Mel@M2‐exos significantly reduced the expression of GRP78, CHOP, and Caspase‐3, suggesting that Mel@M2‐exos could inhibit ER stress signaling pathway activated through high concentrations of LPS, reverse cell apoptosis of hPDLCs, and improve their impaired osteogenic differentiation. Taken together, M2‐exos, in concert with melatonin, can mediate immune reprogramming, inhibit overactivated ER stress and UPR, rescue osteogenic, cementogenic, and odontogenic differentiation of hPDLCs under inflammatory conditions and promote macrophages repolarization in order to restore the periodontal microenvironment and ultimately accelerate periodontal tissue regeneration.

Nevertheless, several practical issues, such as safe exosome manufacturing and quality control, must be addressed before they can be used in the clinic.^[^ ^43^ ^]^ We also consider that many engineered M2‐exos are required to achieve good therapeutic results, and scalable mass‐production solutions are needed.^[^ ^44^ ^]^ Therefore, there is a need to develop optimized technologies to facilitate clinical use of engineered exosomes for the delivery of chemicals and genes in the future.

## 4 Conclusion

It is of great importance to achieve bone regeneration in periodontitis by eliminating chronic inflammation and promoting osteogenesis of hPDLCs. Macrophages are critical regulators of periodontal inflammation and tissue repair, which produce exosomes containing characteristic contents associated with parent cells and have immunomodulatory effects. As a unique class of natural nanocarriers, exosomes avoid clearance by the mononuclear phagocyte system and enhance nanoparticle accumulation in target tissues compared to complex cell‐mediated drug delivery systems or toxic synthetic nanocarriers. Melatonin, a class of natural hormones produced via the pineal gland, is osteoprotective and promotes the regeneration of inflamed periodontal bone, whereas the biological mechanisms involved are not yet precise. In this article, we successfully constructed melatonin‐loaded engineered M2 macrophage‐derived exosomes by ultracentrifugation and cyclic sonication, which can effectively target inflammatory regions and mediate immune reprogramming to promote macrophage repolarization. At the same time, the continual release of melatonin from the natural nanoparticles, in concert with M2‐exos, reduced the excessive ER stress and UPR, subsequently inhibited cell apoptosis, and promoted the restoration of the osteogenic, cementogenic, and odontogenic differentiation of hPDLCs under inflammatory state. Further, Mel@M2‐exos accelerated periodontal regeneration in rats with ligation‐induced periodontitis.

## 5 Experimental Section

### Cells Isolation and Culture

The RAW 264.7 macrophage cell line and primary hPDLCs were used. The RAW 264.7 cells were acquired from American Type Culture Collection (ATCC, Manassas, USA). According to previous experimental instructions, the primary hPDLCs were isolated from human periodontal ligament tissue.^[^ ^45^ ^]^ The experimental protocol and surgical operation were approved by the Clinical Ethics Committee of the Ninth People's Hospital of Shanghai Jiao Tong University (SH9H‐2017‐204). Briefly, the premolars extracted for orthodontic treatment from patients aged 12–15 years were collected and rinsed thrice with PBS (HyClone, Logan, USA) containing 5% penicillin/streptomycin (HyClone). Then, the periodontal membrane was scraped off with a sterile blade and cut into small pieces, which were seeded in a cell culture dish covered with coverslips. These cells were both cultured in Dulbecco's modified Eagle's medium (DMEM) (HyClone) containing 10% fetal bovine serum (FBS) (Gibco, Thermo Fisher Scientific, Waltham, USA) and 1% penicillin/streptomycin at 37 °C in 5% CO_2._ The hPDLCs between passages 2 and 6 were used for subsequent studies.

### Exosomes Isolation and Characterization

To obtain M2‐exos, RAW 264.7 cells were seeded and allowed to become 70–80% confluent, then interleukin‐4 (IL‐4; Invitrogen, CA, USA) (100 ng mL^−1^) was added to the medium to differentiate primary macrophages into M2‐type macrophages, after 24 h, they were incubated in medium with exosome‐free 10% FBS (System Bioscience, CA, USA). The conditioned medium from M2 macrophages was ultrafiltered through a molecular weight cut‐off (MWCO) of 200 kDa to concentrate the exosome‐containing solution. The supernatant was collected in a centrifuge tube and centrifuged at 1000 g for 30 min to remove dead cells, then at 10 000 g for 30 min to remove cell debris. The resulting supernatant was ultracentrifuged at 100 000 g for 90 min to pellet exosomes, which were suspended in PBS, and ultracentrifuged at 100 000 g for 60 min to wash away impurities. Purified exosomes were resuspended in PBS containing 50 × 10^−3^ m trehalose and stored at −80 °C for further characterization. All procedures were carried out at 4 °C.

The exosome concentration was measured with a bicinchoninic acid (BCA) Protein Assay Kit (Beyotime Biotechnology, Haimen, China). Western blot was conducted to examine the exosome markers. The morphology of M2‐exos and Mel@M2‐exos were assessed with TEM (JEOL, Tokyo, Japan). In brief, exosomes were loaded into a copper grid for 3–5 min. After staining with 2% (w/v) phosphotungstic acid for 2–3 min, the exosomes were examined by TEM. Further, the particle size distribution and concentration were identified by nanoparticle tracking analysis (NTA) with a NanoSight NS300 instrument (Malvern, Worcestershire, UK).

### Labeling and Internalization of Exosomes

M2‐exos were labeled with fluorescent 3,3′‐dioctadecyloxacarbocyanine perchlorate (DiD; Invitrogen) according to the manufacturer's recommendations. Briefly, purified M2‐exos were incubated in 5 × 10^−6^ m DiD for 15 min at 37 °C and were then ultracentrifuged at 100 000 g for 90 min to remove unbound dye. After being washed twice in PBS with centrifugation at 100 000 g, the labeled exosomes were resuspended in PBS before use. hPDLCs seeded on 24‐well plates were incubated at 37 °C with DiD‐labeled M2‐exos. Uptake was stopped after 4 h by washing. Then, the cells were fixed in 4% paraformaldehyde (PFA) for 30 min and permeabilized in 0.5% Triton‐X (Beyotime Biotechnology) in PBS for 15 min. Samples were stained sequentially with Actin‐Tracker Green (Beyotime Biotechnology) for 30 min and 4′,6‐diamidino‐2‐phenylindole (Beyotime Biotechnology) for 5 min at room temperature.

### Mel Release Studies

In vitro drug release studies were performed at 37 °C simulating human body temperature using PBS (pH = 7.4) as the releasing agent. Samples (Mel@M2‐exos) were first placed in dialysis bags and subsequently 1 mL of sample was withdrawn at predetermined time points, followed by the addition of an equal volume of sample to the release medium to continue the drug release. The concentration of drug in the samples was determined using a UV spectrophotometer (UV‐2000, LANGUAGES, FRANKVILI, WI).

### Cell Viability Assay

The cell vitality was detected using a CCK‐8 kit (Beyotime Biotechnology). The experiments were performed on days 1, 4, and 7 to evaluate the effect of M2‐exos with different melatonin concentrations on the proliferation of hPDLCs. Briefly, the culture solution was aspirated from the well plates and washed with PBS to remove apoptotic cells and cellular metabolites. A total of 200 µL of the working solution was added to each well and incubated with the cells for 4 h, then 100 µL of the working solution was aspirated, the supernatant was transferred to a 96‐well plate, and the absorbance of each sample supernatant was measured at 450 nm using an enzyme marker.

### Quantitative Real‐Time Polymerase Chain Reaction (qRT‐PCR)

The hPDLCs were incubated in medium for 7 days before adding 1 mL RNAiso plus (Invitrogen), resuspended, and transferred to a 1.5 mL RNA‐free centrifuge tube. After lysis on ice for 5 min, the chloroform was added, shaken upside down, and centrifuged. After centrifugation, the upper layer of liquid was aspirated, an equal volume of isopropanol was added, mixed well and centrifuged after 10 min at 4 °C. The supernatant was removed and dried naturally for 5–10 min until the white mRNA precipitate becomes clear. The mRNA concentration of each sample was measured after dissolution in RNase‐free water. The mRNA concentration was adjusted using the primary script RT kit (Takara, Tokyo, Japan), and a 20 µL system of reverse transcription reaction solution was prepared and reverse transcribed to obtain cDNA samples. qPCR was performed using the TB green premix Ex Taq kit (Takara) to prepare the reaction system in a fluorescent qPCR instrument (Roche LightCycler 96, Basel, Switzerland). The internal reference group was GADPH. Table S1 (Supporting Information) shows the sequences of the qPCR primers (Shanghai Sangon Biotechnology, Shanghai, China).

### Western Blot Analysis

The hPDLCs cultured in different exosomes were collected on day 7, 100 µL of RIPA buffer (Beyotime Biotechnology) containing protease inhibitors (Beyotime Biotechnology) was added, resuspended, lysed on ice, and then centrifuged for 20 min. The BCA method determined the extracted protein concentrations, which was adjusted by adding appropriate amounts of PBS and SDS‐PAGE protein buffer (Beyotime Biotechnology). The samples were boiled in a 97 °C water bath for 15 min and stored at −80 °C. A preformed gel plate was mounted, 5 µL of preset protein marker (Epizyme Biomedical Technology, Shanghai, China) was added as a reference for molecular protein weight, and samples were added for electrophoresis. After completion of electrophoresis, the film was removed and immersed in transfer solution for 5 min and transferred at a constant flow of 300 mA for 1 h. After removing the protein transfer NC film (Bio‐Rad, CA, USA), it was closed with 5% w/v skimmed milk (Beyotime Biotechnology) at room temperature 1 h. The primary antibody (Table S2, Supporting Information) was incubated overnight at 4 °C on a shaker. The secondary antibody (Table S2, Supporting Information) was incubated at room temperature for 1 h. Chemiluminescent substrates for HRP detection dipped in a biomolecular imager.

### Immunofluorescence (IF) Staining

In brief, the medium was removed along the edge of the 24‐well plate and hPDLCs were washed thrice with PBS. The cells were fixed with 4% paraformaldehyde at room temperature for 15 min, then cleaned with PBS thrice. 0.1% Triton X‐100/PBS (PBST, Beyotime Biotechnology) solution was soaked for 5 min at room temperature to permeate the cell membrane. Sealed with 1% w/v BSA/PBS at room temperature for 30 min. Then a sufficient amount of diluted primary antibody (Table S2, Supporting Information) was added dropwise and placed in a wet box and incubated overnight at 4 °C. The next day, diluted Alexa Fluor Plus 647 secondary antibody (Table S2, Supporting Information) was added dropwise and incubated in a wet box at 37 °C for 1 h. Finally, 4′,6‐diamidino‐2‐phenylindole was diluted with PBS at a ratio of 1:500, and the nuclei were stained at room temperature for 5 min and rinsed with PBS for 3 times. A small amount of anti‐fluorescence quenching agent was added to hPDLCs, and CLSM (LEXT OLS5100, OLYMPUS, Japan) was shot.

### Alkaline Phosphatase Staining and Activity

ALP staining (Beyotime Biotechnology) was performed on day 7 of the cell culture. The hPDLCs were fixed with 4% paraformaldehyde for 15 min, incubated for 45 min at 37 °C with the addition of staining solution, and excess dye was washed with distilled water. After drying, the staining results were observed under an inverted light microscope and photographed. ALP activity detection kit (Jiancheng Technology, Nanjing, China) was used to determine its activity. The cells were lysed on ice with lysis buffer, and the supernatant was collected after centrifugation. The total protein amount of each group of cells was determined by the BCA method, and the total amount of protein contained in each group of cells was calculated according to the BSA standard curve. The ALP activity of the cells was normalized to the total amount of protein in each group, and the ALP OD value/total protein concentration was calculated.

### Alizarin Red Staining

After day 21 of osteogenesis induction, ARS was used to observe the formation of mineralized nodules. Briefly, cells were fixed in 4% paraformaldehyde for 30 min and washed thrice with PBS. ARS solution (Beyotime Biotechnology) was added and incubated at 37 °C for 45 min. The excess staining solution was then removed with distilled water. After drying, the cells were observed under an inverted light microscope and photographed.

### Animals

All experimental protocols, surgery operations, and postoperative antibiotics were approved by the Animal Ethics committee of the Shanghai Ninth People's Hospital Affiliated to Shanghai Jiaotong University (HKDL‐2017‐339). A rat ligature periodontitis model was used to evaluate the preventive and therapeutic effects of M2‐exos and Mel@M2‐exos on inflammatory periodontal bone loss.^[^ ^46^ ^]^ Briefly, 20 6‐week‐old male SD rats were purchased and randomly divided into four groups after 1 week of adaptive rearing, and the rats were anesthetized by intraperitoneal injection of sodium pentobarbital (Merck Millipore, Darmstadt, Germany). Periodontitis was induced by tying a 5‐0 ligature wire in the cervical region of the maxillary bilateral first molars. GelMA hydrogel loading with 200 µL M2‐exos and Mel@M2‐exos (or 0.9% NaCl solution as control and GelMA hydrogel groups) was injected into the palatal gingiva of the ligated molars every 2 days at the proximal, middle, and distal sites, respectively. Two weeks later, all rats were executed by overdose anesthesia. Tissues were collected for the following analyses.

The upper jaw was scanned using micro‐CT (skscan 1176, Bruker Micro‐CT, Kontich, Belgium) to observe changes in alveolar bone morphology, quantity and quality. Using Ctvox and DataViewer software, three‐dimensional (3D) digital images of the different areas and cross‐sectional images in the proximal‐distal and buccolingual directions were reconstructed and obtained. Bone correlation parameters such as bone volume (BV), tissue volume (TV), and BV/TV were also measured around the ligated molar (CTAn software). The measuring bone‐related parameters was the bone area around the maxillary first molars, excluding the teeth. The bone height between maxillary first and second molars in different groups analyzed by micro‐CT (Image‐pro‐plus 6.0 software).

### Histological Analysis

For histological analysis, the maxillae of experimental rats were fixed in 10% neutral buffered formalin solution and decalcified in 0.5 m EDTA for 8 weeks. Tissue sections of the distal mesial side were also made according to the above method. H&E (Solarbio, Beijing, China), Masson (Solarbio) staining, and specific anti‐TRAP; Jiancheng Technology) staining were then used to evaluate the histological alterations and osteoclast activity of the periodontal tissue. The number of osteoclasts per square millimeter of the alveolar bone surface was counted and analyzed according to the TARP‐stained images. The distances of cemento‐enamal junction to alveolar bone crest (CEJ–ABC) were measured to assess bone loss. The expression of GRP78, CHOP, Caspase, CEMP‐1, and DSPP was measured via the immunohistochemical method. Then the quantitative analysis was achieved with Image Pro Plus 6.0 software.

### Statistical Analysis

All quantitative data were performed as the mean ± SD. The statistical analysis was calculated by one‐way analysis of variance (ANOVA) followed by Turkey's multiple comparison tests or Student's *t*‐test via GraphPad Prism 7.0 software. The sample size (*n*) for each statistical analysis has been reported in the corresponding “figure legends.” Single analysis of variance at *p* < 0.05 was regarded as statistical significance. The label * and # were utilized to represent statistical significance.

## Conflict of Interest

The authors declare no conflict of interest.

## Supporting information

Supporting Information

## Data Availability

The data that support the findings of this study are available from the corresponding author upon reasonable request.
