# Supplementary material for: Melatonin Engineering M2 Macrophage‐Derived Exosomes Mediate Endoplasmic Reticulum Stress and Immune Reprogramming for Periodontitis Therapy
Source: Adv Sci (Weinh). 2023 Jul 14;10(27):2302029. doi: 10.1002/advs.202302029 (PMC10520618; doi:10.1002/advs.202302029)
Supplement: Supplementary file 1 — Supporting Information [file ADVS-10-2302029-s001.pdf]

ADVANCED SCIENCE
Open Access

Supporting Information

for *Adv. Sci.*, DOI 10.1002/advs.202302029

Melatonin Engineering M2 Macrophage-Derived Exosomes Mediate Endoplasmic Reticulum Stress and Immune Reprogramming for Periodontitis Therapy

Ya Cui, Shebin Hong, Yunhui Xia, Xiaojing Li, Xiaoya He, Xiangying Hu, Yaxin Li, Xudong Wang, Kaili Lin* and Lixia Mao**

Supporting Information

Melatonin engineering M2 macrophage-derived exosomes mediate endoplasmic reticulum stress and immune reprogramming for periodontitis therapy

Ya Cui[a1], Shebin Hong[a1], Yunhui Xia[a1], Xiaojing Li[a], Xiaoya He[a], Xiangying Hu[a], Yaxin Li[a], Xudong Wang[a*], Kaili Lin[a*], Lixia Mao[a*]

[a] Department of Oral & Cranio-Maxillofacial Surgery, Shanghai Ninth People's Hospital, College of Stomatology, Shanghai Jiao Tong University School of Medicine; National Clinical Research Center for Oral Diseases; Shanghai Key Laboratory of Stomatology & Shanghai Research Institute of Stomatology, Shanghai 200011, China

[1] These three authors contributed equally to this work.

[*]Corresponding authors：

Lixia Mao

Department of Oral & Cranio-Maxillofacial Surgery, Shanghai Ninth People's Hospital, College of Stomatology, Shanghai Jiao Tong University School of Medicine; National Clinical Research Center for Oral Diseases; Shanghai Key Laboratory of Stomatology & Shanghai Research Institute of Stomatology, Shanghai 200011, China

E-mail: Maolx1302@126.com

Kaili Lin

Department of Oral & Cranio-Maxillofacial Surgery, Shanghai Ninth People's Hospital, College of Stomatology, Shanghai Jiao Tong University School of Medicine; National Clinical Research Center for Oral Diseases; Shanghai Key Laboratory of Stomatology & Shanghai Research Institute of Stomatology, Shanghai 200011, China

E-mail: lklecnu@aliyun.com

Xudong Wang

Department of Oral & Cranio-Maxillofacial Surgery, Shanghai Ninth People's Hospital, College of Stomatology, Shanghai Jiao Tong University School of Medicine; National Clinical Research Center for Oral Diseases; Shanghai Key Laboratory of Stomatology & Shanghai Research Institute of Stomatology, Shanghai 200011, China

E-mail: wang70@hotmail.com

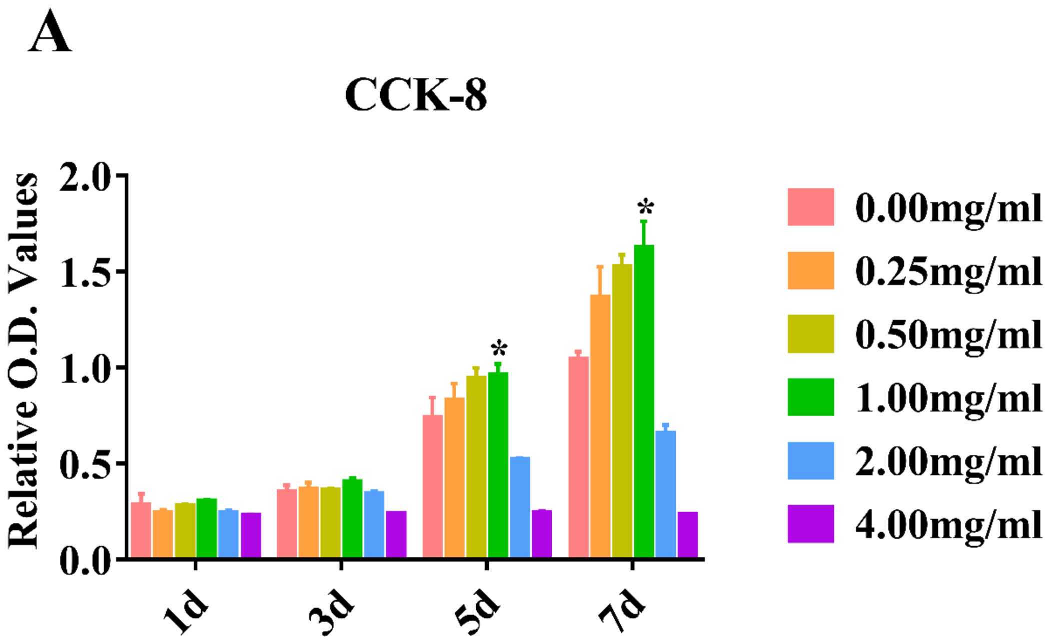

Figure S1. Gating strategies for the concentration screening of melatonin. (A) Gating strategy to detect the optimal melatonin concentration loaded in M2-exos for the proliferation of hPDLCs. Data represented as mean ± SD (n=3). The significance of the data was calculated by the one-way ANOVA.

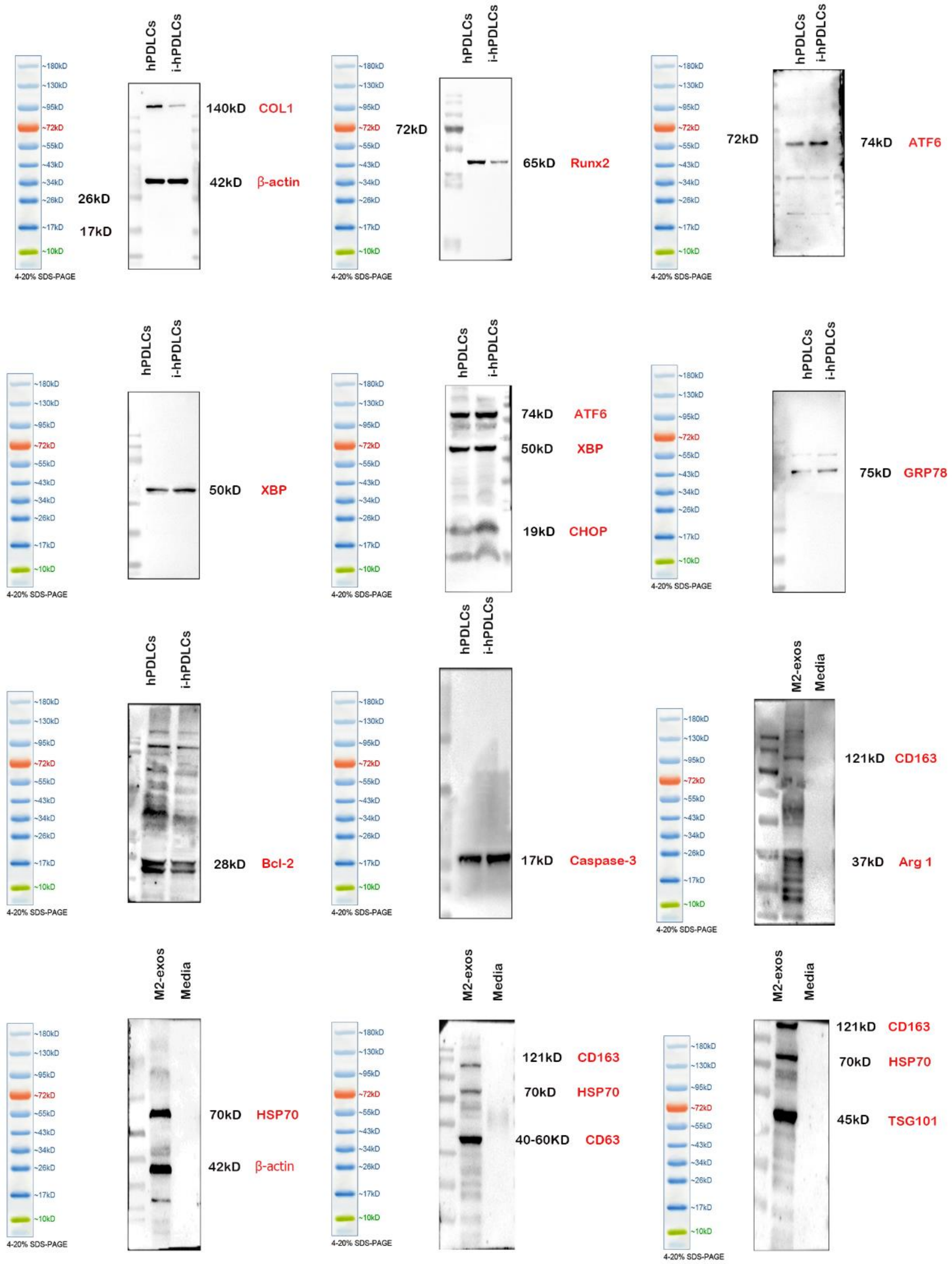

Figure S2. The raw, unmodified uncropped scanning figures of western blot.

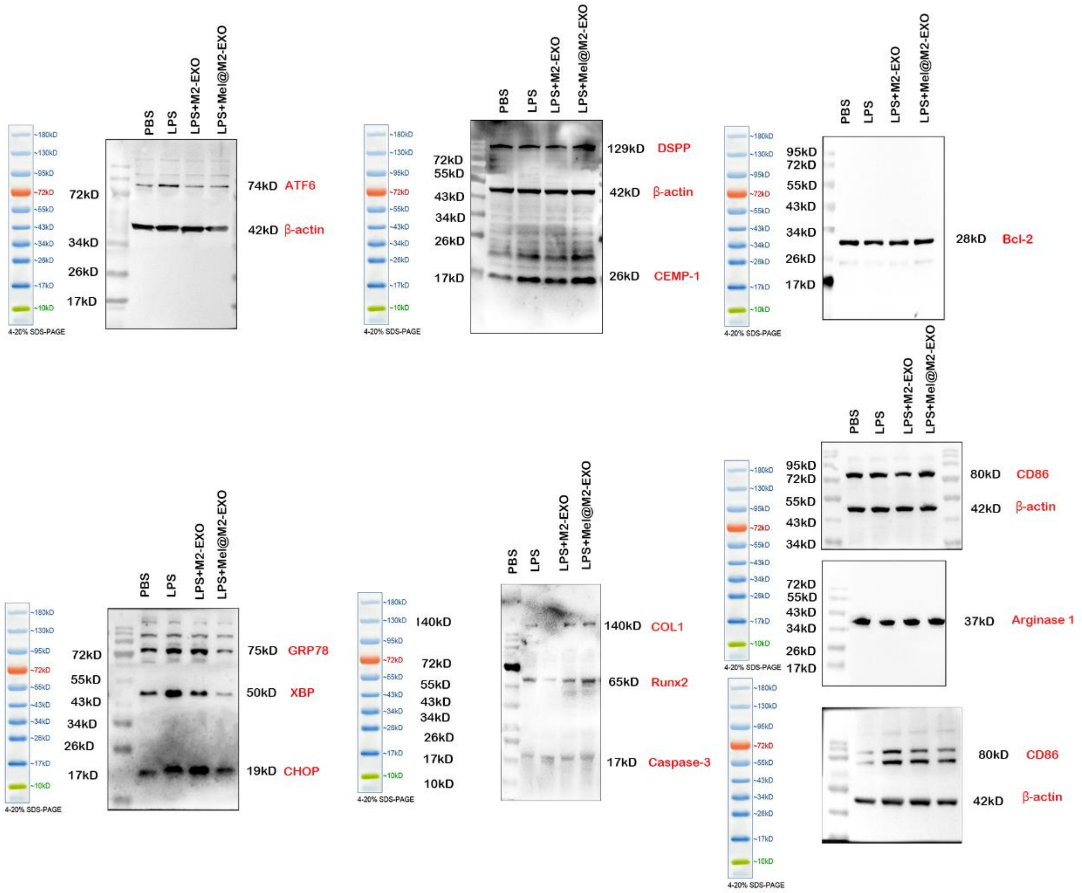

Figure S3. The raw, unmodified uncropped scanning figures of western blot.

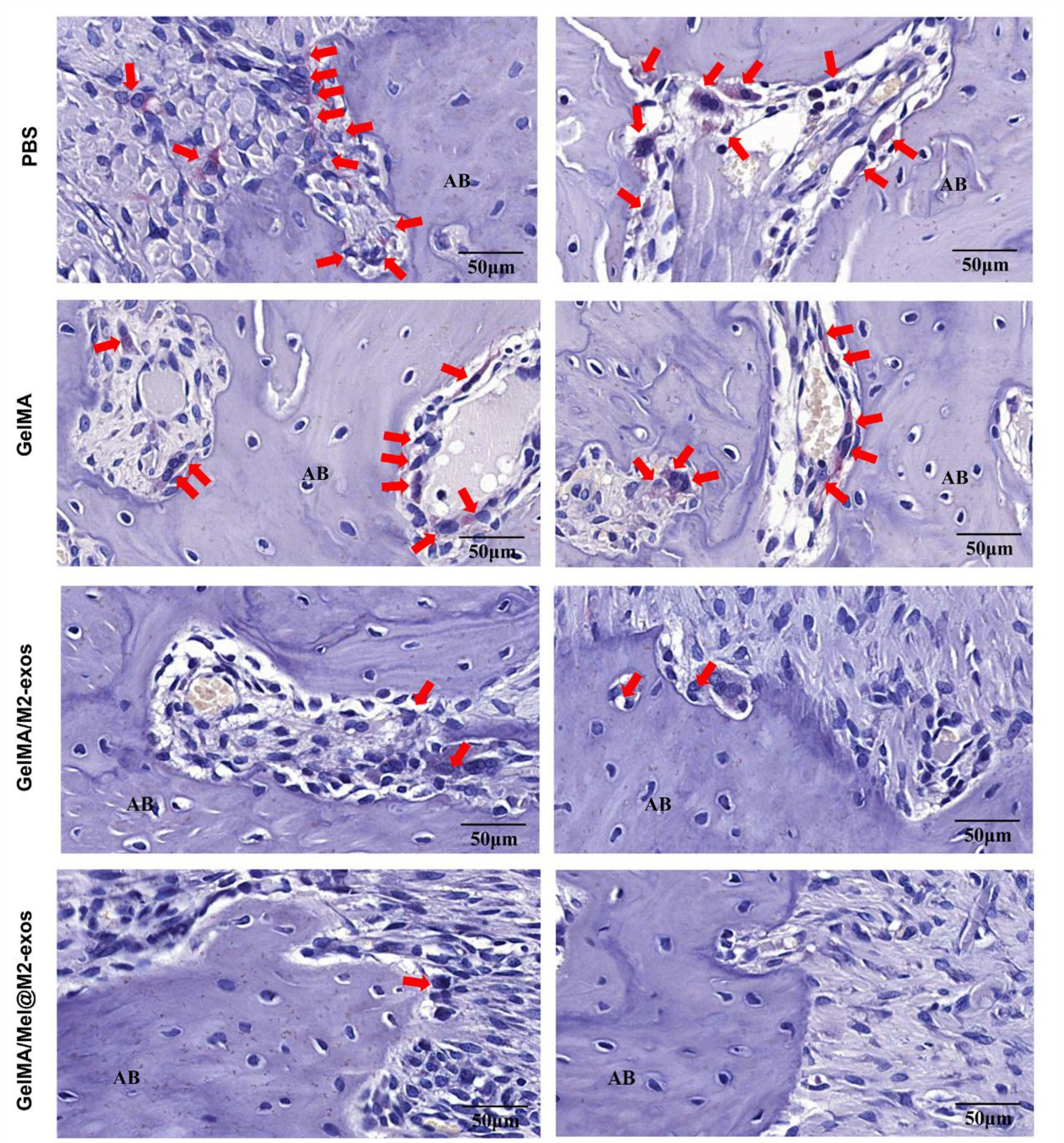

Figure S4. TRAP staining images (arrows indicate osteoclasts that are dyed red) of maxillary alveolar bone in rat ligature-induced periodontitis model.

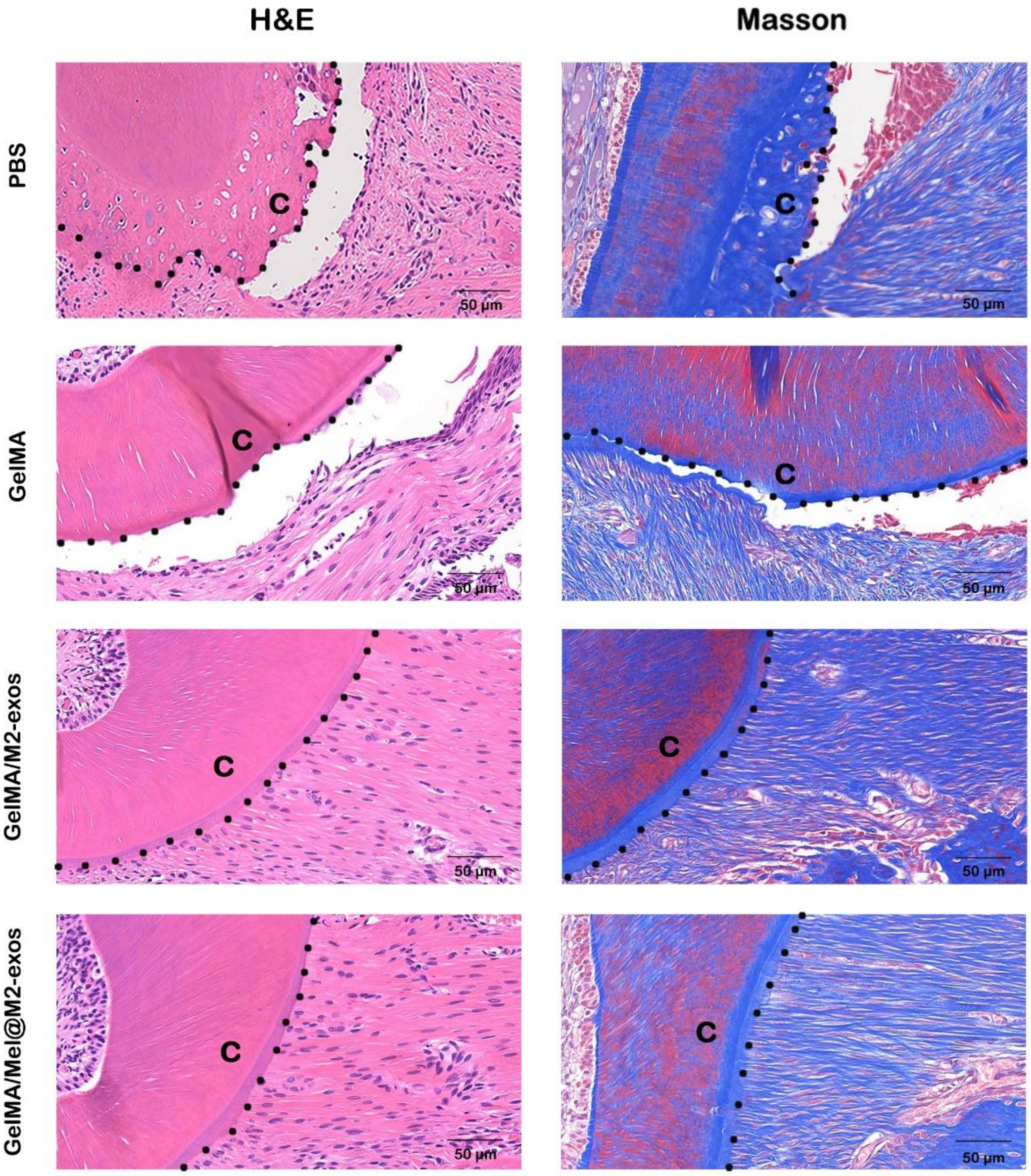

Figure S5. H&E and Masson staining. C = cementum.

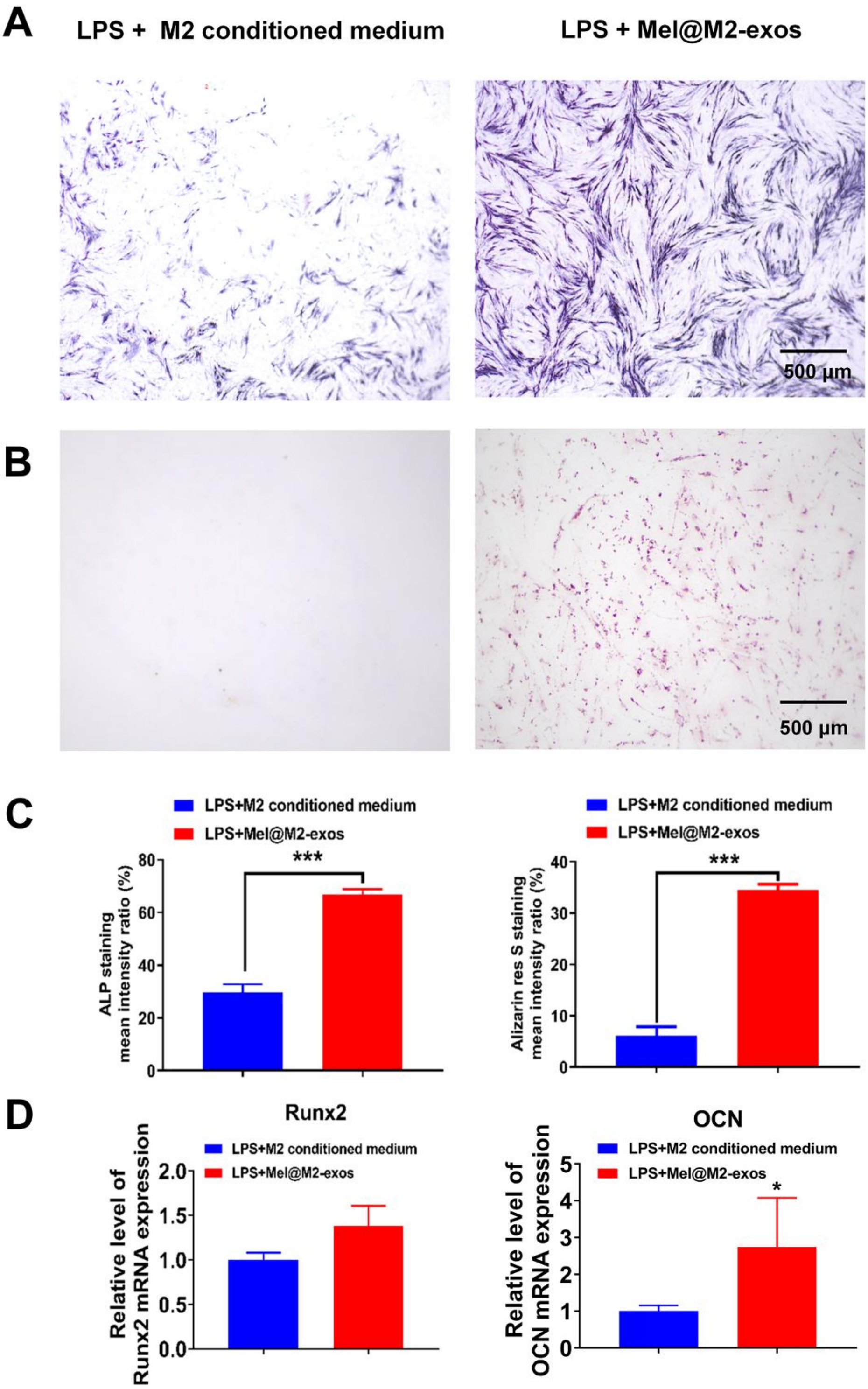

Figure S6. Comparison of the effects of M2 macrophage conditioned medium and Mel@M2-exos on osteogenic differentiation of inflammatory hPDLCs. (A) ALP staining of inflammatory hPDLCs cultured with M2 conditioned medium and Mel@M2-exos (inverted microscope; scale

bar: 500 μm). (B) ARS and calcium deposition production of inflammatory hPDLCs cultured with M2 conditioned medium and Mel@M2-exos (inverted microscope; scale bar: 500 μm). (C) The quantitative analysis of ALP staining, ARS and calcium deposition production in inflammatory hPDLCs cultured with M2 macrophage conditioned medium and Mel@M2-exos. (D) The mRNA levels of osteogenic genes in inflammatory hPDLCs cultured with M2 macrophage conditioned medium and Mel@M2-exos, including Runx2 and OCN. Data represented as mean ± SD (n=3). The significance of the data was calculated by the one-way ANOVA.

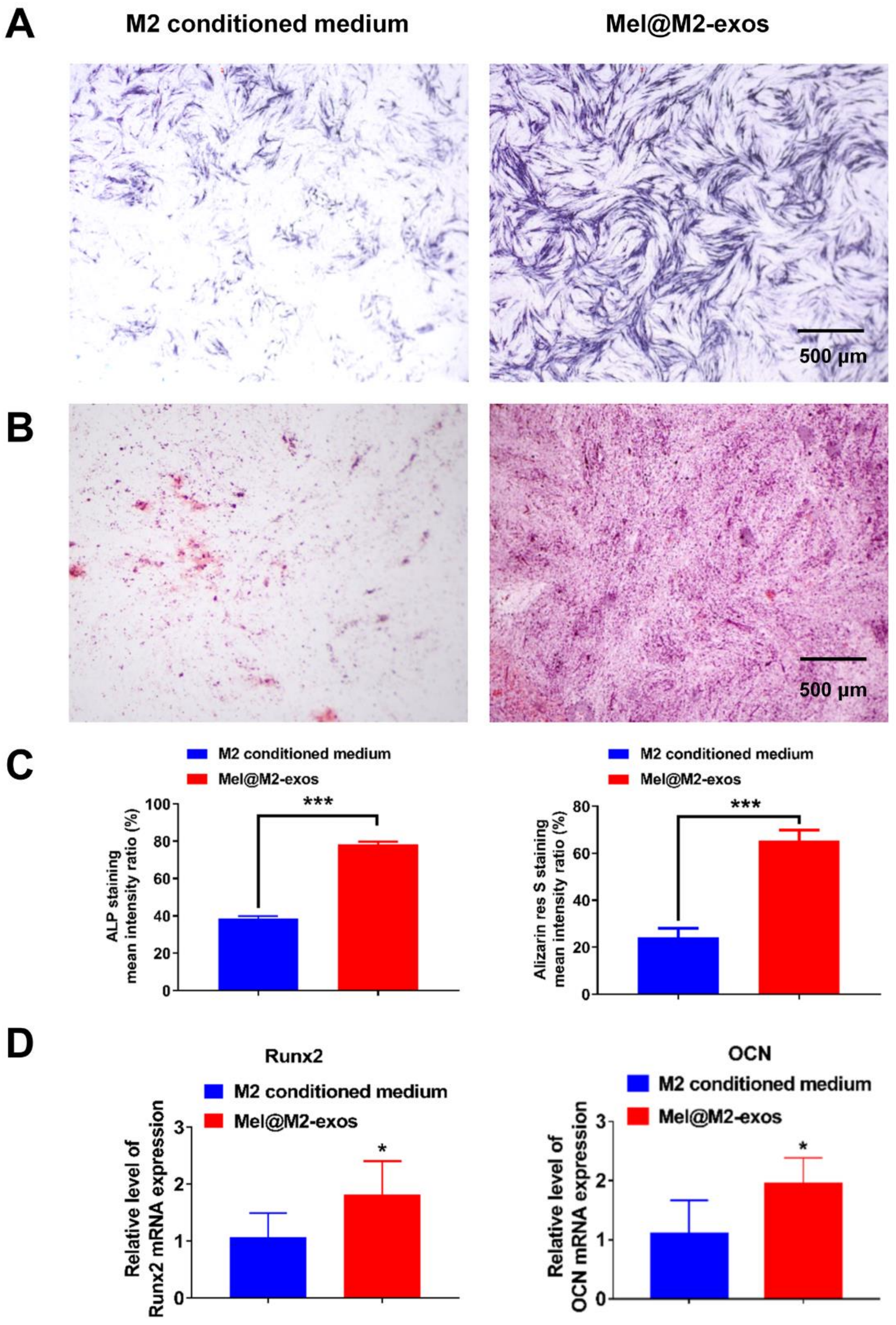

Figure S7. Comparison of the effects of M2 macrophage conditioned medium and Mel@M2-exos on osteogenic differentiation of human periodontal ligament cells (hPDLCs). (A) ALP staining in M2 conditioned medium and Mel@M2-exos (inverted microscope; scale bar: 500 μm). (B) ARS and calcium deposition production in M2 conditioned medium and Mel@M2-exos (inverted microscope; scale bar: 500 μm). (C) The quantitative analysis of ALP staining, ARS and calcium

deposition production in hPDLCs cultured with M2 macrophage conditioned medium and Mel@M2-exos. (D) The mRNA levels of osteogenic genes, including Runx2 and OCN. Data represented as mean ± SD (n=3). The significance of the data was calculated by the one-way ANOVA.

Table S1. Primers used in this study

Gene	Forward and reverse sequences (5′-3′)
GAPDH-F (H)	CGCTCTCTGCTCCTCCTGTT
GAPDH-R (H)	CCATGGTGTCTGAGCGATGT
ALP-F (H)	GACCCTTGACCCCCACAAT
ALP-R (H)	GCTCGTACTGCATGTCCCCT
COL1-F (H)	AGAACAGCGTGGCCT
COL1-R (H)	TCCGGTGTGACTCGT
OCN-F (H)	GGCAGCGAGGTAGTGAAGAG
OCN-R (H)	GATGTGGTCAGCCAACTCGT
RUNX-2-F (H)	GGAGTGGACGAGGCAAGAGTTT
RUNX-2-R (H)	AGCTTCTGTCTGTGCCTTCTGG
CAP-F (H)	CTGCGCGCTGCACATGG
CAP-R (H)	GCGATGTCGTAGAAGGTGAGCC
CEMP1-F (H)	GGGCACATCAAGCACTGACAG
CEMP1-R (H)	CCCTTAGGAAGTGGCTGTCCAG
DSPP-F(H)	TTTGGGCAGTAGCATGGGC
DSPP-R(H)	CCATCTTGGGTATTCTCTTGCCT
DMP1-F(H)	CTCCGAGTTGGACGATGAGG
DMP1-R(H)	TCATGCCTGCACTGTTCATTC
RANKL-F (H)	GGCTCATGGTTAGATCTGGC
RANKL-R (H)	TGACCAATACTTGGTGCTTCC
OPG-F (H)	TCAAGCAGGAGTGCAATCG
OPG-R (H)	AGAATGCCTCCTCACACAGG
GRP78-F (H)	TCAAGTTCTTGCCGTTCAAGG
GRP78-R (H)	AAATAAGCCTCAGCGGTTTCTT
CHOP-F (H)	CAAGAGGTCCTGTCTTCAGATGA
CHOP-R (H)	TCTGTTTCCGTTTCCTGGTTC
ATF6-F (H)	TGCTTTACATTCCTCCAC
ATF6-R (H)	TGACTTGGTCCTTTCTACTT
XBP1-F (H)	CCTGGTTGCTGAAGAGGAGG
XBP1-R (H)	CCATGGGGAGATGTTCTGGAG
PERK-F (H)	GTGATAAAGGTTTCGGTTGCTG
PERK -R (H)	TGTTTTCTGTGGCTCCTCTGG

IRE1-F (H)	CCTAGTCAGTTCTGCGTCCG
IRE1-R (H)	TTCCATCCAGCGTTGACACA
IL-1-F (H)	TGGCTTATTACAGTGGCAATGAGGATG
IL-1-R (H)	TGTAGTGGTGGTCGGAGATTCGTAG
IL-6-F (H)	GGTGTTGCCTGCTGCCTTCC
IL-6-R (H)	GTTCTGAAGAGGTGAGTGGCTGTC
TNF-α-F (H)	CGTGGAGCTGGCCGAGGAG
TNF-α-R (H)	AGGAAGGAGAAGAGGCTGAGGAAC
COX-2-F (H)	TGAGCATCTACGGTTTGCTG
COX-2-R (H)	AACTGCTCATCACCCCATTC
CCL-2-F (H)	AGAATCACCAGCAGCAAGTGTCC
CCL-2-R (H)	TCCTGAACCCACTTCTGCTTGG
GAPDH-F (M)	AGGTCGGTGTGAACGGATTTG
GAPDH-R (M)	TGTAGACCATGTAGTTGAGGTCA
iNOS-F (M)	GTTCTCAGCCCAACAATACAAGA
iNOS-R (M)	GTGGACGGGTCGATGTCAC
CD86-F (M)	TGTTTCCGTGGAGACGCAAG
CD86-R (M)	TTGAGCCTTTGTAAATGGGCA
CD206-F (M)	CTCTGTTCAGCTATTGGACGC
CD206-R (M)	CGGAATTTCTGGGATTCAGCTTC
Arginase 1-F (M)	CTCCAAGCCAAAGTCCTTAGAG
Arginase 1-R (M)	AGGAGCTGTCATTAGGGACATC

Table S2. Antibodies used in this study

MARKER (SPECIES)	DILUTION	DISTRIBUTOR/SOURCE
Primary antibodies:		
CD63 Rabbit mAb	1:1000	Abcam, London, UK
HSP70 Rabbit mAb	1:1000	Abcam, London, UK
Anti-TSG101 Rabbit mAb	1:1000	Abcam, London, UK
β-actin Mouse mAb (4F2)	1:1000	Abcam, London, UK
CD163 Rabbit mAb	1:1000	Abcam, London, UK

Arginase 1 Rabbit mAb	1:1000	Cell Signaling, Massachusetts, USA
iNOS Rabbit mAb	1:1000	Cell Signaling, Massachusetts, USA
RUNX2 Rabbit mAb	1:1000	Cell Signaling, Massachusetts, USA
COL1A1 Rabbit mAb	1:1000	Abcam, London, UK
GRP78 BiP Rabbit mAb	1:1000	Abcam, London, UK
ATF6 Rabbit mAb	1:1000	Invitrogen, CA, USA
XBP Rabbit mAb	1:1000	Cell Signaling, Massachusetts, USA
CHOP Rabbit mAb	1:1000	Cell Signaling, Massachusetts, USA
Caspase-3 Rabbit mAb	1:1000	Cell Signaling, Massachusetts, USA
Bcl-2 Rabbit mAb	1:1000	Cell Signaling, Massachusetts, USA
CEMP-1 Rabbit mAb	1:1000	Abcam, London, UK
DSPP Rabbit mAb	1:1000	Abmart, Shanghai, China
Secondary antibodies:		
Anti-mouse IgG HRP-linked Ab	1:1000	Abcam, London, UK
Anti-rabbit IgG HRP-linked Ab	1:1000	Abcam, London, UK
Anti-rabbit IgG (Alexa Fluor™ Plus 647)	1:500	Invitrogen, CA, USA
